# Determination of the Global Pattern of Gene Expression in Yeast Cells by Intracellular Levels of Guanine Nucleotides

**DOI:** 10.1128/mBio.02500-18

**Published:** 2019-01-22

**Authors:** Andy Hesketh, Marta Vergnano, Stephen G. Oliver

**Affiliations:** a Cambridge Systems Biology Centre, University of Cambridge, Cambridge, United Kingdom; b Department of Biochemistry, University of Cambridge, Cambridge, United Kingdom; c School of Pharmacy and Biomolecular Sciences, University of Brighton, Brighton, United Kingdom; Harvard Medical School; University of Tennessee Health Science Center; Chalmers University of Technology

**Keywords:** ATP, GTP, *Saccharomyces cerevisiae*, metabolism, purine metabolism

## Abstract

Correlations between gene transcription and the abundance of high-energy purine nucleotides in Saccharomyces cerevisiae have often been noted. However, there has been no systematic investigation of this phenomenon in the absence of confounding factors such as nutrient status and growth rate, and there is little hard evidence for a causal relationship. Whether transcription is fundamentally responsive to prevailing cellular energetic conditions via sensing of intracellular purine nucleotides, independently of specific nutrition, remains an important question. The controlled nutritional environment of chemostat culture revealed a strong correlation between ATP and GTP abundance and the transcription of genes required for growth. Short pathways for the inducible and futile consumption of ATP or GTP were engineered into S. cerevisiae, permitting analysis of the transcriptional effect of an increased demand for these nucleotides. During steady-state growth using the fermentable carbon source glucose, the futile consumption of ATP led to a decrease in intracellular ATP concentration but an increase in GTP and the guanylate energy charge (GEC). Expression of transcripts encoding proteins involved in ribosome biogenesis, and those controlled by promoters subject to SWI/SNF-dependent chromatin remodelling, was correlated with these nucleotide pool changes. Similar nucleotide abundance changes were observed using a nonfermentable carbon source, but an effect on the growth-associated transcriptional programme was absent. Induction of the GTP-cycling pathway had only marginal effects on nucleotide abundance and gene transcription. The transcriptional response of respiring cells to glucose was dampened in chemostats induced for ATP cycling, but not GTP cycling, and this was primarily associated with altered adenine nucleotide levels.

## Abstract (precis)

This paper investigates whether, independently of the supply of any specific nutrient, gene transcription responds to the energy status of the cell by monitoring ATP and GTP levels. Short pathways for the inducible and futile consumption of ATP or GTP were engineered into the yeast Saccharomyces cerevisiae, and the effect of an increased demand for these purine nucleotides on gene transcription was analyzed. The resulting changes in transcription were most consistently associated with changes in GTP and GEC levels, although the reprogramming in gene expression during glucose repression is sensitive to adenine nucleotide levels. The results show that GTP levels play a central role in determining how genes act to respond to changes in energy supply and that any comprehensive understanding of the control of eukaryotic gene expression requires the elucidation of how changes in guanine nucleotide abundance are sensed and transduced to alter the global pattern of transcription.

## INTRODUCTION

In order for Saccharomyces cerevisiae cells to survive, grow, and proliferate, their metabolism must respond, in both the short term and the long term, to changes in their environment. This is achieved via control exerted at multiple levels, including the regulation of gene transcription and the modulation of gene product activities by both posttranslational modifications and the allosteric binding of metabolites to enzymes and other effectors (1–5). These differing routes of control allow metabolic responses to take place on different time scales: the allosteric and posttranslational mechanisms can produce almost instantaneous effects, while the reprogramming of gene expression produces adjustments over the longer term. The intracellular abundance of small-molecule metabolites plays a key role in these responses not only at the level of enzyme activity but also at the level of the regulation of gene expression. Thus, metabolites can exert their effects via the modulation of TORC1 kinase activity by amino acid availability (6) and through the influence of intracellular cyclic AMP (cAMP) and acetyl-CoA concentrations on the activity of the PKA kinase complex (7, 8) and on histone acetylation (9). It has also been proposed that SNF1 kinase activity is regulated by ADP nucleotide levels (10). Since gene expression also determines metabolite abundance by providing anabolic enzymes, the state of cellular metabolism at any time can be viewed as the product of the interplay between metabolite concentrations and the abundance and activity of gene products.

ATP and GTP, together with the other related nucleotide di- and monophosphates, are among the most highly connected metabolites in the yeast metabolic network (http://yeast.sourceforge.net/), and their intracellular concentrations are a key measure of general metabolic status. The concept of a cellular adenylate energy charge (AEC), defined by the relative concentrations of all three phosphorylated adenosine nucleotides [ATP] + 0.5[ADP]/[ATP] + [ADP] + [AMP], was first proposed in 1967 to describe the energy status of living cells (11), and the homeostatic control of AEC has since become a common paradigm in cell physiology. The analogous cellular guanylate energy charge (GEC) is also considered relevant, based on the requirement for GTP as the immediate source of energy for peptide bond formation in cellular protein synthesis (12).

Many studies of *S*. *cerevisiae* have reported correlations between changes in phosphorylated adenosine and guanosine nucleotide concentrations and changes in cell physiology or gene expression. Batch-grown cultures using glucose as the carbon source exhibit marked decreases in the ratios of both [ATP]/[ADP] and [GTP]/[GDP] on transition from exponential growth to the stationary phase (13). Conversely, the addition of glucose to respiring or glucose-starved cells produced marked increases in these ratios (13, 14). *In silico* studies have also predicted a role for [GTP]/[GDP] in modulating the activity of the PKA complex by influencing the activity state of the Ras2p GTP-binding protein responsible for controlling production of the upstream cAMP signal (15–17).

Perhaps the most convincing *in vivo* data has been collected from yeast cultures as they transit through cycles of respiratory oscillations where expression of a majority of genes in the genome becomes highly synchronized (18, 19). These oscillations, characterized by alternating periods of high and low oxygen consumption (oxidative and reductive phases, respectively), are associated with corresponding oscillations in [ATP]/[ADP] ratios and with a periodic large-scale rewiring of gene expression such that energy-intensive processes (such as ribosome biogenesis) are implemented during the periods of high respiratory activity/energy production (18–22). Extensive nucleosome repositioning occurs as the yeast cells transit through a cycle, and modulation of the activity of ATP-dependent chromatin remodelling complexes has been proposed to contribute to the transcriptional reprogramming by making core promoter elements of target genes more accessible for use (20–22). Acetyl-CoA could be a key metabolic signal through its indirect effect on the acetylation status of histones and other proteins involved in chromatin remodelling (9, 23). Conversely, a direct role for ATP has also been proposed, since many chromatin remodelling complexes are dependent on this nucleotide for their activity (20).

All these observations provide circumstantial evidence for the yeast cell transcriptional program being, in some way, generally responsive to changing nucleotide levels. They suggest a cycle of regulation between metabolic activity, nucleotide concentrations, and gene expression. Evidence for a causal relationship is, however, sparse since confounding variables such as growth rate (24) or nutritional status (25) cannot be adequately controlled when experiments are performed in batch culture. In order to circumvent these difficulties, we have developed strains of S. cerevisiae in which an increased use of ATP or GTP can be induced in cells independently of the external nutrient supply or changes in the rate of growth. This has been achieved by engineering yeast to express enzymes from bacteria that enable the futile consumption of either ATP or GTP. Analysis of the transcriptional response following induction of these ectopic pathways during continuous culture in chemostats demonstrates that inducing the futile consumption of ATP, but not GTP, results in a major reprogramming of gene transcription in yeast cells grown on a fermentative carbon source (glucose) but not on those cells growing by respiration on acetate. This genetic reprogramming does not require changes to the AEC but, instead, is associated with an increase in GTP and the GEC. Induction of the futile consumption of ATP, but not GTP, also has an effect on the transcriptional response of respiring cells to glucose repression.

## RESULTS

### Exploiting bacterial enzymes to engineer the futile consumption of ATP or GTP in S. cerevisiae.

**FIG 1:**
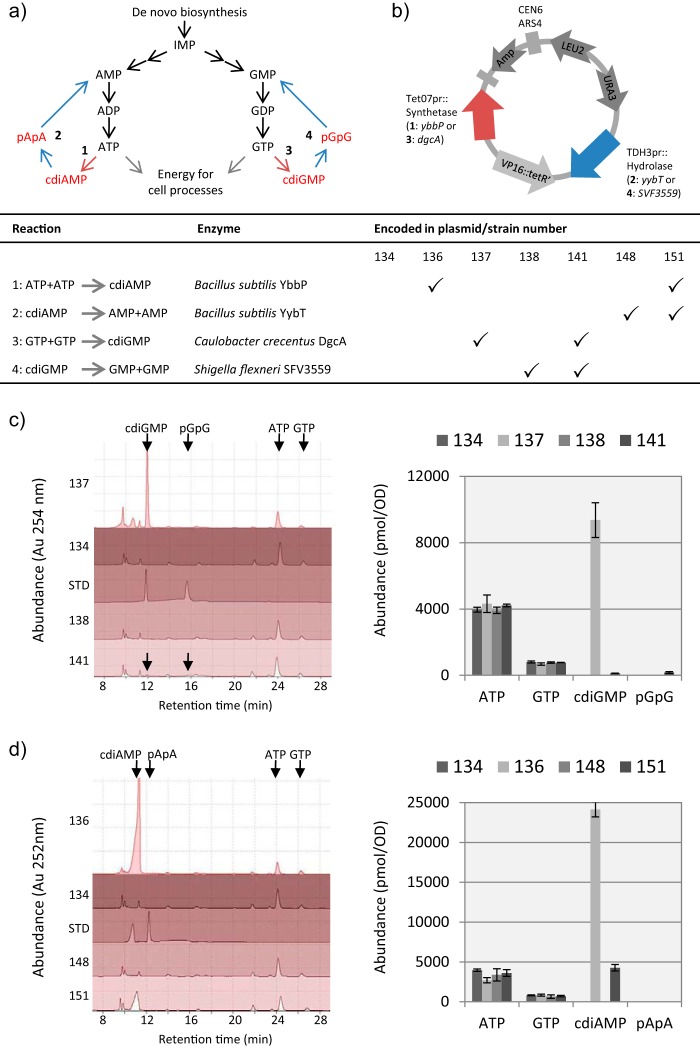
Engineering inducible consumption of ATP or GTP in S. cerevisiae using coding sequences from bacteria. Shunt pathway loops for the futile turnover of each nucleotide triphosphate to their corresponding monophosphates via cyclic dinucleotide monophosphate (cdiNMP) intermediates (a) were introduced into S. cerevisiae BY4741-112 on a series of related centromeric plasmids (b). A targeted HPLC-UV metabolomics analysis (c and d) of the constructed strains following induction of expression of the plasmid genes encoding the cdiNMP synthetase enzymes using doxycycline (DOX) (5 μg/ml) shows high-level production of cdiGMP (c) (strain 137) and cdiAMP (d) (strain 136) compared to the control strain carrying the empty vector (c and d) (strain 134). Constitutive coexpression of appropriate cdiNMP hydrolase genes completes the pathway loop and removes the accumulation of cdiGMP (c) (strain 141) and cdiAMP (d) (strain 151) during similar inductions. None of the bacterial metabolites was detected in strains constitutively expressing the cdiNMP hydrolase genes alone (strain 138 [c] and strain 148 [d]). In both panels c and d, the panels to the left are overlays of HPLC-UV chromatograms (arbitrary absorbance units [Au] with detection at 254 nm) from a representative analysis of metabolite extracts from cultures of the strains indicated. Analysis of a mixed standard (STD) containing 2,000 pmol of either cdiGMP and pGpG (c) or cdiAMP and pApA (d) indicates the retention times of these metabolites. The panels to the right show normalized nucleotide abundances (picomoles/optical density) calculated from the HPLC-UV data as an average of duplicate experiments. Values are means ± standard deviations (error bars).

To generate strains of S. cerevisiae where a direct and specific increase in the consumption of ATP or GTP can be controllably induced, we heterologously expressed genes encoding bacterial enzymes that use these nucleotides as substrates (Fig. 1 and Table 1). The doxycycline (DOX)-inducible expression of a cyclic dinucleotide monophosphate (cdiNMP) synthetase enzyme was coupled with the constitutive expression of its corresponding hydrolase to create short pathways for the futile degradation of two molecules of ATP or GTP to two molecules of AMP or GMP via their respective cdiNMP intermediates (Fig. 1a). Expression constructs were based on a centromeric plasmid carrying two nutritional marker genes, *URA3* and *LEU2*, to enable selection for the continuous presence of the plasmid during prolonged culture (Fig. 1b). A repressor construct to minimize transcription from the DOX-inducible promoter in the absence of inducer (26) was also integrated into the genome of the host strain BY4741. This combination of nutritional markers on the plasmid generated prototrophic strains (Table 1) capable of growing on a minimal medium containing only a suitable carbon source, ammonium sulfate, potassium phosphate, and trace essential vitamins and minerals (27). This eliminates confounding effects on the yeast transcriptional program arising from the metabolic consequences of auxotrophy (28).

**TABLE 1:** Plasmids and yeast strains used in this study

Plasmid or strain	Description or relevant genotype	Reference
Plasmids		
pAH112	pRS-*HIS3*-*MET15* TetR::*SSN6*	36
pAH134	pRS-*LEU2*-*URA3* VP16::TetR' TetO_7_pr	36
pAH136	pRS-*LEU2*-*URA3* VP16::TetR' TetO_7_pr::*ybbP*	36
pAH137	pRS-*LEU2*-*URA3* VP16::TetR' TetO_7_pr::*dgcA*	36
pAH138	pRS-*LEU2*-*URA3* VP16::TetR' TetO_7_pr:: TDH3pr::*SFV3559*	This work
pAH141	pRS-*LEU2*-*URA3* VP16::TetR' TetO_7_pr::*dgcA* TDH3pr::*SFV3559*	This work
pAH148	pRS-*LEU2*-*URA3* VP16::TetR' TetO_7_pr:: TDH3pr::*yybT*	This work
pAH151	pRS-*LEU2*-*URA3* VP16::TetR' TetO_7_pr::*ybbP* TDH3pr::*yybT*	This work

Strains		
BY4741	*MAT****a*** *ura3*Δ0 *leu2*Δ0 *met15*Δ0 *his3*Δ1	
BY4741-112	BY4741 with integrated plasmid pAH112 (*his3*Δ1::pAH112 *ura3*Δ0 l*eu2*Δ0)	36
BY4741-112-134	BY4741-112 with centromeric plasmid pAH134 (prototroph)	36
BY4741-112-136	BY4741-112 with centromeric plasmid pAH136 (prototroph)	36
BY4741-112-137	BY4741-112 with centromeric plasmid pAH137 (prototroph)	36
BY4741-112-138	BY4741-112 with centromeric plasmid pAH138 (prototroph)	This work
BY4741-112-141	BY4741-112 with centromeric plasmid pAH141 (prototroph)	This work
BY4741-112-148	BY4741-112 with centromeric plasmid pAH148 (prototroph)	This work
BY4741-112-151	BY4741-112 with centromeric plasmid pAH151 (prototroph)	This work

The functionality of the engineered pathways was assessed by analysis of the intracellular nucleotide composition in the host strains following induction with DOX during growth in batch culture (Fig. 1c and d). Strain BY4741-112-137 carrying only the inducible cdiGMP synthetase gene variant *dgcA0244* from the bacterium Caulobacter crescentus (29) showed intracellular production of cdiGMP to levels approximately 10 times the concentration observed for GTP and twice that for ATP, the latter being the most abundant natural intracellular nucleotide in yeast cells (Fig. 1c). Similar DOX-induced expression of the cdiAMP synthetase gene *ybbP* from Bacillus subtilis (30) yielded ca. six times more intracellular cdiAMP than ATP (Fig. 1d; strain BY4741-112-136). In each case, incorporation of genes determining cdiNMP hydrolase activity in the strains prevented accumulation of the heterologous cdiNMP metabolites, indicating a successful conversion of the nucleotide triphosphate to monophosphate via the cdiNMP intermediate. Thus, coexpression of the cdiGMP hydrolase gene *SVF3559* from Shigella flexneri (31) in strain BY4741-112-141 reduced cdiGMP production by 98.5% (Fig. 1c). Only low levels of the linear dinucleotide pGpG were observed, consistent with the conversion of the majority of the cdiGMP generated by the synthetase DgcA to GMP. Coexpression of a portion of the Bacillus subtilis cdiAMP hydrolase gene *yybT* (32) with the DOX-inducible *yybP* gene in strain BY4741-112-151 reduced cdiAMP production by 82.5% compared to expression of *ybbP* alone (Fig. 1d).

### Using carbon-limited chemostat cultures to analyze the effect of inducing increases in ATP or GTP consumption during steady-state growth.

Aerobic cultures of S. cerevisiae have the potential to generate energy from externally supplied carbon sources by either fermentative or respiratory pathways. In order to analyze the effect of inducing increases in ATP or GTP consumption in cells using these different routes for energy production, strains were grown in chemostats at dilution rates of 0.11 to 0.12 h^−1^ under carbon-limited conditions using a defined minimal medium containing either 0.5% (wt/vol) acetate (respiratory) or 0.25% (wt/vol) glucose (respiratory) as the energy source. Cultures were sampled to analyze any changes in the intracellular nucleotide pool composition and genome-wide transcript abundance, as illustrated in Fig. S1 in the supplemental material and described in detail in Materials and Methods. Chemostat cultivation, characterized by growth at a fixed rate in constant nutritional conditions, was used to control for confounding effects of any changes in growth rate or external nutrient supply during induction. Each culture was sampled during steady-state growth in the noninducing conditions (designated SS1) and 3 h, 6 h, and 9 h after inducing the heterologous pathway by adding DOX (designated T1, T2, and T3, respectively), and during steady-state growth in the induced cultures (designated SS2). To investigate any effect on the transition from respiratory to fermentative growth, the chemostats respiring acetate were additionally treated with a pulse of glucose (final concentration, 1.6% [wt/vol]) after collecting the SS2 sample, and further samples were taken 15 min (G1), 30 min (G2) and 60 min (G3) after treatment. Transcription of the transgenes in the chemostat experiments proceeded as designed, and production of the cyclic and linear dinucleotide intermediates was within the range expected from the batch culture data in Fig. 1 (Fig. S1). The genes encoding the hydrolase enzymes ranked in the top 2% most highly expressed genes, and the induced expression of the synthetase constructs was in the top 1%. Normalized transcriptome and nucleotide abundance data are provided in Data Set S1, and nucleotide abundance plots are provided in Data Set S2.

10.1128/mBio.02500-18.1 FIG S1 Induction of the futile ATP-cycling or GTP-cycling pathways during steady-state growth in carbon-limited chemostat cultures using sodium acetate (a) or glucose (b) as the energy source. Triplicate chemostats containing control strains (light gray curves) or NTP-cycling strains (dark gray curves) were grown to an initial steady state (SS1) prior to induction by the addition of 5 mg/ml DOX. Samples (indicated by the dotted lines) for transcriptome and targeted metabolome analyses were taken 3 h (T1), 6 h (T2), and 9 h (T3) after induction and when a new postinduction steady state had been reached (SS2). In the acetate growth experiment, chemostats were also treated with a pulse of glucose (final concentration, 1.6% [wt/v]) after collecting the SS2 sample, and further samples were taken 15 min (G1), 30 min (G2), and 60 min (G3) after treatment. For clarity, only the G3 sample is indicated above. The effect on biomass in the chemostats was followed by off-line measurements of the culture absorbance at 600 nm (OD600nm). Shading indicates the 95% confidence interval for loess-fitting smoothed curves to the data points. Strains used for the GTP- and ATP-cycling experiments were as follows: for GTP-cycling experiments, the control strain was BY4741-112-138 and the NTP-cycling strain was BY4741-112-141; for the ATP-cycling experiments, the control strain was BY4741-112-148 and the NTP-cycling strain was BY4741-112-151. (c) Transcript abundance profiles for the nucleotide-cycling pathway genes during the chemostat runs illustrated are presented in panels a and b. As expected, SVF3559 is constitutively highly expressed in strains BY4741-112-138 (138) and BY4741-112-141 (141), while transcription of *dgcA* is induced only after DOX addition to the BY4741-112-141 chemostat. Similarly, for the ATP-cycling chemostats, *yybT* is constitutively expressed in both strains BY4741-112-148 (148) and BY4741-112-151 (151), and the *ybbP* gene encoding the cdiAMP synthetase is induced only in strain BY4741-112-151. Download FIG S1, EPS file, 3.5 MB. Copyright © 2019 Hesketh et al. 2019 Hesketh et al. This content is distributed under the terms of the Creative Commons Attribution 4.0 International license.

10.1128/mBio.02500-18.7 DATA SET S1 Normalized transcript abundance and intracellular nucleotide abundance data for the triplicate chemostat experiments. Download Data Set S1, XLSX file, 16.7 MB. Copyright © 2019 Hesketh et al. 2019 Hesketh et al. This content is distributed under the terms of the Creative Commons Attribution 4.0 International license.

10.1128/mBio.02500-18.8 DATA SET S2 Plots of the changes in the abundances of intracellular nucleotides detected during the chemostat experiments. Download Data Set S2, PDF file, 0.1 MB. Copyright © 2019 Hesketh et al. 2019 Hesketh et al. This content is distributed under the terms of the Creative Commons Attribution 4.0 International license.

### Nucleotide indicators of high cellular energy status correlate with transcription of genes required for ribosome biogenesis.

**FIG 2:**
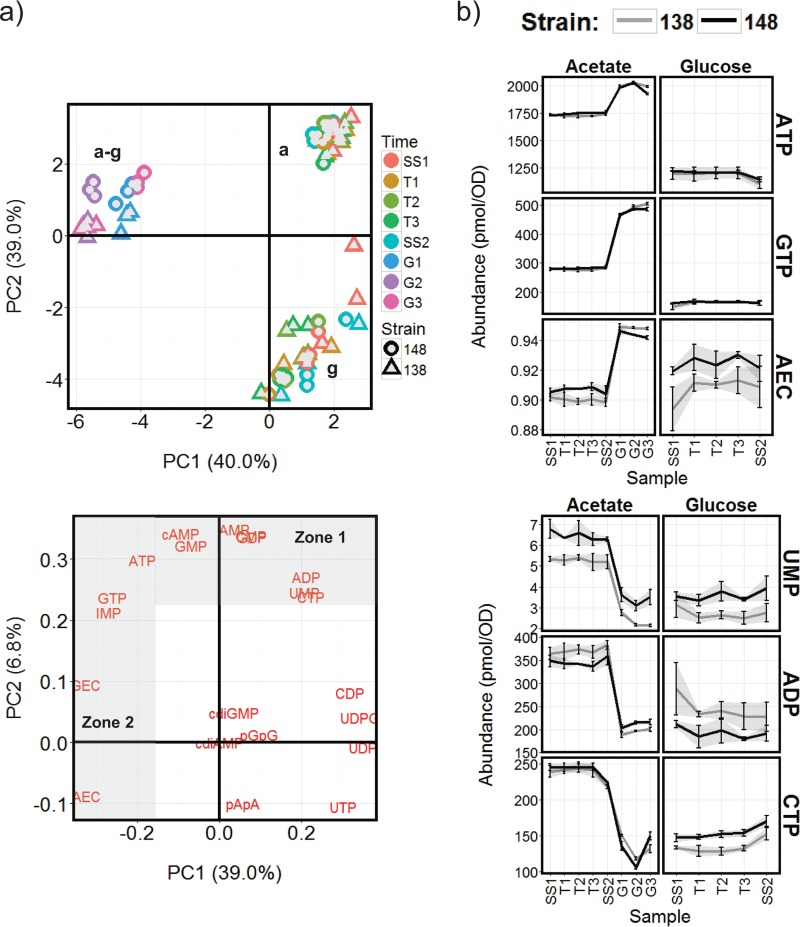
Changes in intracellular nucleotide concentrations during the control chemostat cultures using strains BY4741-112-138 (138) and BY4741-112-148 (148) and either acetate or glucose as the carbon source. (a) PCA analysis summarizing the differences in nucleotide content of cells grown in the different chemostat conditions. The scores plot (top left panel) shows distinct grouping of the acetate (a), glucose (g), and glucose-treated acetate (a-g) chemostat samples, while the loadings plot (bottom left panel) highlights the underlying differences in nucleotide abundances. Nucleotide abundances distinguishing group a from group g are in the shaded zone 1, and those separating group a-g from group a or g are in zone 2. (b) Abundance profiles for example nucleotides from these shaded zones are provided to the right (see Data Set S2 in the supplemental material for all profiles).

**FIG 3:**
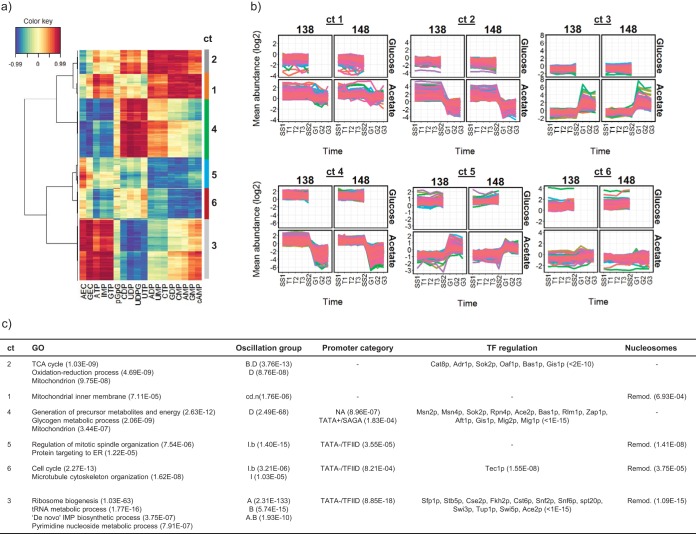
Correlations between gene transcription and intracellular nucleotide abundance in the control chemostat cultures using strains BY4741-112-138 (138) and BY4741-112-148 (148). (a) Clustered image map from sPLS analysis of nucleotide (*x*-axis) and transcript (*y*-axis) abundance data showing the most highly correlated nucleotide and transcript pairings, clustering 1,609 transcripts into six cluster groups (ct) (sPLS threshold of 0.8). (b) Abundance profiles for the transcripts in groups 1 to 6 in samples SS1-SS2 (glucose chemostats) and SS1-G3 (acetate chemostats). (c) Functional classification of genes in cluster groups (ct) 1 to 6 using GO enrichment analysis (only headline significant GO terms are shown); comparison with the consensus respiratory oscillation cluster group membership from Machne and Murray (22) (A, A.B, and B = anabolic, oxidative; D = catabolic, reductive; see reference 22 for other group definitions); analysis of transcription initiation promoter category as reported in Rhee and Pugh (34) (TATA−, TATA-less; TATA+, TATA-containing; TFIID, TAF1-enriched; SAGA, TAF1-depleted; NA, not assigned); transcription factor (TF) regulation predictions (yeastract database [35]); and comparison to the promoter nucleosome dynamics category assigned in Nocetti et al. (21) (for nucleosomes, static = dyad range 0 in reference 21; Remod.= dyad range ≥5 in reference 21). The numbers in brackets are significance *P* values from Fisher’s exact tests for category enrichment. Complete GO and yeastract analysis results are provided in Data Set S3.

To define the basal relationship between nucleotide and transcript abundances in the experimental system, covariance in the sets of data obtained for the control chemostat strains BY4741-112-138 and BY4741-112-148 was analyzed using principal components analysis (PCA) (Fig. 2) and by sparse partial least-squares (sPLS) canonical analysis using the mixOmics R package (33) (Fig. 3 and Data Set S3). PCA of the nucleotide abundance data (Fig. 2a) indicates that the cultures growing in acetate are distinguished from those grown in glucose by higher levels of ATP, GTP, IMP, cAMP, AMP, GMP, CMP, GDP, ADP, UMP, and CTP (nucleotides shown in zone 1 of Fig. 2a), but they exhibit similar values for AEC and GEC. The addition of glucose to the acetate-grown cultures changes the levels of zone 1 nucleotides to levels more similar to those observed in the glucose-grown chemostats, with the notable exception of IMP, GTP, and ATP which, in contrast, are increased further (Fig. 2 and Data Set S2). Both AEC and GEC were markedly higher in these samples compared to either the steady-state acetate- or glucose-limited chemostat samples.

10.1128/mBio.02500-18.9 DATA SET S3 sPLS canonical analysis looking for correlations between the transcript abundance and nucleotide abundance data matrices, as also illustrated in Fig. 3. Download Data Set S3, XLSX file, 4.8 MB. Copyright © 2019 Hesketh et al. 2019 Hesketh et al. This content is distributed under the terms of the Creative Commons Attribution 4.0 International license.

Interestingly, all the indicators of high cellular energy status (i.e., AEC, GEC, ATP, GTP, and IMP [IMP being a key intermediate in *de novo* purine nucleotide biosynthesis]) show a strong positive correlation with a cluster of transcripts encoding proteins significantly enriched in functions associated with the biogenesis of the translational machinery and with those proteins involved in purine and pyrimidine nucleotide biosynthesis (cluster 3 in Fig. 3). This cluster shows significant similarity to the cell growth and anabolic supercluster genes (A, A.B, and B) previously identified as being expressed in the oxidative phase of a respiratory oscillation cycle (22) (Fig. 3c). Conversely, a general negative correlation was observed between the high-energy nucleotides and transcription associated with the generation of precursor metabolites and energy, glycogen metabolism, and the mitochondrion (cluster 4 in Fig. 3), and this cluster exhibits a significant similarity to the reductive phase supercluster (D) of a respiratory oscillation (22). The abundances of CDP, UDP, UDPG, and UTP exhibit a strong positive correlation with transcript cluster 4, which is characterized by similar expression levels between the acetate and glucose steady-state growth conditions, but marked repression on the addition of glucose to the cultures growing on acetate as the principal carbon source (Fig. 3a). Transcript clusters 1 and 2 also exhibit repression following this glucose treatment, but they tend to be more highly expressed during steady-state growth on acetate than on glucose and correlate most highly with cAMP and the lower-energy nucleotide monophosphates plus ADP, GDP, and CTP. These clusters are enriched for functions associated with the TCA cycle, oxidation-reduction, and the mitochondrion (Fig. 3). Thus, this analysis suggests a distinction between the repression of reserve energy metabolism correlated with the higher-energy uridine nucleotides on the one hand (cluster 4), and the downregulation of mitochondrial respiration correlated with low-energy nucleotides on the other hand (clusters 1 and 2).

### Gene transcription positively correlating with the abundance of high-energy nucleotides tends to occur from promoters found in remodellable chromatin locations.

Nocetti et al. (21) observed that the dynamic range in the transcription of genes during a respiratory oscillation cycle is closely related to the extent of repositioning of +1 nucleosomes taking place at their promoters, and also to the use of the transcriptional coactivators SAGA or TFIID. Thus, in their experimental system, acute nucleosome remodelling occurred preferentially at SAGA promoters and facilitated dynamic changes in the expression of genes required for growth. Snf2p, the ATP-dependent catalytic subunit of the SWI/SNF chromatin-remodelling complex, was identified as playing a fundamental role in both the nucleosome repositioning and growth-associated gene expression. To determine whether the changes in gene expression identified in the chemostat growth conditions used in this study are associated with any particular type of promoter, we tested the transcription clusters identified above (Fig. 3a and b) for significant overrepresentation of control by the remodellable promoters as defined by Nocetti et al. (21) and for previously published associations with TFIID or SAGA (34) (Fig. 3c). Transcript cluster 3, which positively correlates with the high-energy nucleotide markers (ATP, GTP, GEC, AEC, and IMP) was found to be significantly enriched for genes transcribed from remodellable promoters, and also for promoters which are both TATA-less and TFIID dominated (Fig. 3c). In contrast, cluster 4, showing a negative correlation, is enriched for TATA-containing SAGA-dominated promoters, and also promoters not assigned to any category by Rhee and Pugh (34), and which do not show enrichment for the remodellable class of promoters (Fig. 3c). Interestingly, analysis of the promoters controlling transcription in cluster 3 using yeastract (35) (Data Set S3) identified significant enrichment for regulation by the TORC1-dependent transcriptional activator Sfp1p and by the SWI/SNF complex (Snf6p, Snf2p, and Swi3p) and its associated recruitment factor Swi5p. Regulation of cluster 4 is predicted to be dominated by the general stress response transcription factors Msn2p/Msn4p and Sok2p (Fig. 3c).

### Induction of ATP cycling increases transcription from promoters regulated by SWI/SNF during steady-state growth on glucose, but not on acetate.

**FIG 4:**
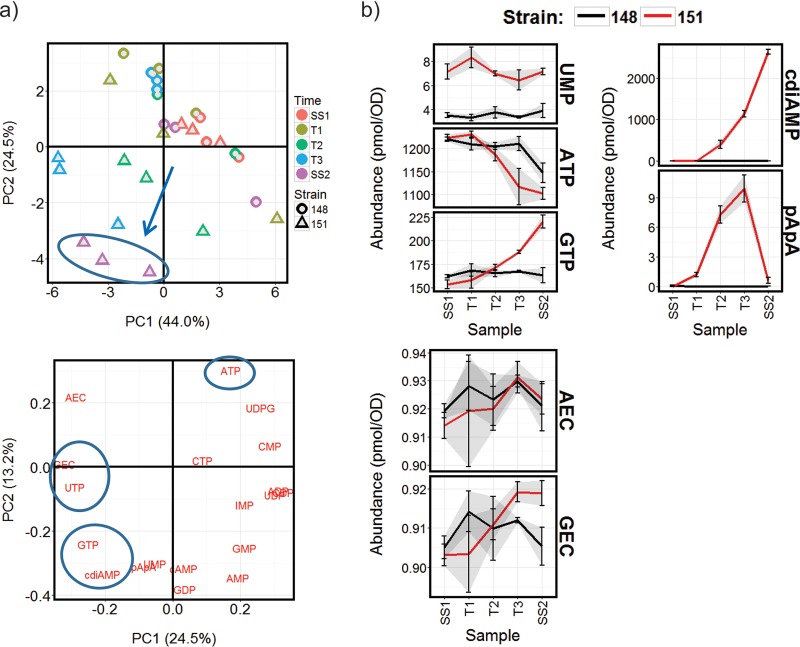
Induction of the ATP-cycling pathway during steady-state growth on glucose produces changes in both ATP and GTP abundance. (a) PCA of normalized nucleotide abundances (scores and loadings plots) identify the changes taking place that distinguish the induced ATP-cycling chemostats, which are illustrated in detail in panel b). All nucleotide abundance profiles are available in Data Set S2.

**FIG 5:**
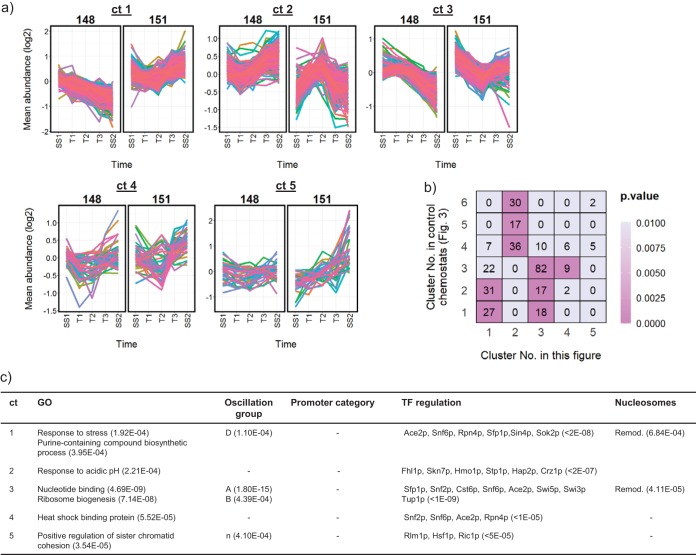
Induction of the ATP-cycling pathway during steady-state growth on glucose leads to coherent changes in gene expression. (a) Transcripts identified as being significantly differently expressed between chemostats of the control strain BY4741-112-148 (148) and the ATP-cycling strain BY4741-112-151 (151) were clustered (Data Set S4), and (b) the clusters were tested for significant overlap with those identified in Fig. 3. The grid presents the number of transcripts shared between each pairwise comparison of clusters, and the color corresponds to the *P* value of a Fisher exact test analyzing the enrichment. (c) Clusters were further functionally characterized as described in the legend to Fig. 3c (Data Set S4).

During growth on glucose, a total of 944 transcripts were identified as being significantly changed in their abundance profiles following induction of the ATP-cycling pathway in strain BY4741-112-151 relative to the control (Data Set S4). Changes in the abundances of intracellular nucleotides taking place during this time period are summarized in Fig. 4; they indicate a general decrease in abundance of ATP but an increase in GTP and GEC. Hierarchical clustering of the transcript abundance data identified two clusters of genes whose expression increased at the point where changes in ATP and GTP abundance, and in GEC, are detected in strain BY4741-112-151 during the transition from SS1 to SS2 (clusters 1 and 3 in Fig. 5). Strikingly, the transcripts in cluster 3 encode proteins that are enriched for functions associated with nucleotide binding (GO:0000166 4.69E-09) and ribosome biogenesis (GO:0042254 7014E-08), and there is a significant overlap between the members of this cluster and transcript cluster 3, which correlates with the high-energy nucleotides in the control chemostat analysis in Fig. 3 (82 transcripts, *P* = 1.01E-47; Fig. 5b). Transcription factor predictions indicate significant regulation of the transcripts in this cluster by the SWI/SNF chromatin remodelling complex (Fig. 5c). It is also enriched for genes exhibiting dynamic nucleosome repositioning during the respiratory oscillation cycle and shows significant overlap with the cell growth and anabolic supercluster genes (A, A.B, and B) in this cycle. No particular enrichment for SAGA- or TFIID-dependent promoters was observed.

10.1128/mBio.02500-18.10 DATA SET S4 Analysis of transcripts identified as being significantly differently expressed following induction of the ATP-cycling or GTP-cycling pathways during the chemostat growth experiments reported in Fig. 5 and 7 and Fig. S2, S3, S4, and S5. Download Data Set S4, XLSX file, 14.1 MB. Copyright © 2019 Hesketh et al. 2019 Hesketh et al. This content is distributed under the terms of the Creative Commons Attribution 4.0 International license.

10.1128/mBio.02500-18.2 FIG S2 Changes in transcript (a) and nucleotide abundance (b and c) following induction of the ATP-cycling pathway during steady-state growth on acetate. Transcripts identified as being significantly differently expressed between chemostats of the control strain BY4741-112-148 (148) and the ATP-cycling strain BY4741-112-151 (151) were clustered (a) (Data Set S4), and the clusters were characterized as described in the legend to Fig. 3 (d) (Data Set S4). PCA of normalized nucleotide abundances (b) (scores and loadings plots) identify the changes taking place that distinguish the induced ATP-cycling chemostats, which are illustrated in detail in panel c. All nucleotide abundance profiles are available in Data Set S2. Download FIG S2, EPS file, 3.5 MB. Copyright © 2019 Hesketh et al. 2019 Hesketh et al. This content is distributed under the terms of the Creative Commons Attribution 4.0 International license.

10.1128/mBio.02500-18.3 FIG S3 Induction of the GTP-cycling pathway has only limited effects on the changes in transcription that occur following the addition of glucose to carbon-limited chemostat cultures growing at steady state on acetate. Transcripts identified as being significantly differently expressed between chemostats of the control strain BY4741-112-138 (138) and the ATP-cycling strain BY4741-112-141 (141) were clustered (a) (Data Set S4), and the clusters were characterized as described in the legend to Fig. 3 (d) (Data Set S4). PCA of normalized nucleotide abundances (b) (scores and loadings plots) identifies the changes taking place that distinguish the induced GTP-cycling chemostats and the glucose-supplemented conditions, which are illustrated in detail in panel c. All nucleotide abundance profiles are available in Data Set S2. Download FIG S3, EPS file, 3.3 MB. Copyright © 2019 Hesketh et al. 2019 Hesketh et al. This content is distributed under the terms of the Creative Commons Attribution 4.0 International license.

10.1128/mBio.02500-18.4 FIG S4 Changes in transcript (a) and nucleotide abundance (b and c) following induction of the GTP-cycling pathway during steady-state growth on glucose. Transcripts identified as being significantly differently expressed between chemostats of the control strain BY4741-112-138 (138) and the GTP-cycling strain BY4741-112-141 (141) were clustered (a) (Data Set S4), and the clusters were characterized as described in the legend to Fig. 3 (d) (Data Set S4). PCA of normalized nucleotide abundances (b) (scores and loadings plots) identify the changes taking place that distinguish the induced GTP-cycling chemostats, which are illustrated in detail in panel c. All nucleotide abundance profiles are available in Data Set S2. Download FIG S4, EPS file, 3.3 MB. Copyright © 2019 Hesketh et al. 2019 Hesketh et al. This content is distributed under the terms of the Creative Commons Attribution 4.0 International license.

10.1128/mBio.02500-18.5 FIG S5 Changes in transcript (a) and nucleotide abundance (b and c) following induction of the GTP-cycling pathway during steady-state growth on acetate. Transcripts identified as being significantly differently expressed between chemostats of the control strain BY4741-112-138 (138) and the GTP-cycling strain BY4741-112-141 (141) were clustered (a) (Data Set S4), and the clusters were characterized as described in the legend to Fig. 3 (d) (Data Set S4). PCA of normalized nucleotide abundances (b) (scores and loadings plots) identify the changes taking place that distinguish the induced GTP-cycling chemostats, which are illustrated in detail in panel c. All nucleotide abundance profiles are available in Data Set S2. Download FIG S5, EPS file, 3.5 MB. Copyright © 2019 Hesketh et al. 2019 Hesketh et al. This content is distributed under the terms of the Creative Commons Attribution 4.0 International license.

The abundance of GTP increases by 50% from SS1 to SS2 following induction of the ATP-cycling pathway, and there is an increase in the GEC (Fig. 4). ATP, however, notably decreases during this period, accompanied by an increase in cdiAMP, but the AEC is maintained. Therefore, there is a common correlation between the abundance of the transcripts shared between cluster 3 in Fig. 5 and cluster 3 in Fig. 3 only with GTP and GEC. Consistent with the increase in GTP and cdiAMP, the transcription of four genes (*ADE1*, *ADE6*, *ADE2*, and *ADE5,7*) encoding enzymes for *de novo* IMP biosynthesis is upregulated following the induction, together with *IMD2*, *GUA1*, and *ADE12* encoding enzymes for the conversion of IMP to GMP or AMP (Data Set S4). Transcript cluster 1 is, in fact, enriched for purine-containing compound biosynthesis (GO:0072522 3.95E-04), and there is evidence for its regulation by SWI/SNF from remodellable promoters (Fig. 5c). This cluster is also enriched for stress response genes and for genes with functions in the cell periphery and phosphate metabolism (Data Set S4). Genes whose transcription decreased relative to the control, particularly from T2 onwards, are found in cluster 2 but are not significantly enriched for any notable functions.

Induction of the futile consumption of ATP in cells grown in carbon-limited chemostats using acetate as the carbon source generated changes in nucleotide composition similar to those observed on glucose (increased cdiAMP, GTP, and GEC and decreased ATP) but did not result in the same changes in gene transcription (Fig. S2 and Data Set S4). Induction of ATP cycling led to a ca. 50% reduction in biomass in the acetate-limited chemostats on transition from SS1 to SS2 (Fig. S1), but this was not accompanied by marked changes in transcription of growth-related genes. Of the 525 differentially expressed transcripts identified, 299 correspond to antisense transcripts (cluster 2 in Fig. S2). GO analysis indicates enrichment for functions associated with respiration encoded by the corresponding genes on the sense strand (GO:0005739 mitochondrion 1.48E-06; GO:0055114 oxidation-reduction process 1.33E-06). Whether the suggested interference in respiration by antisense transcription is related to the observed reduction in biomass is unknown.

### The transcriptional response to glucose is dampened by futile ATP consumption.

**FIG 6:**
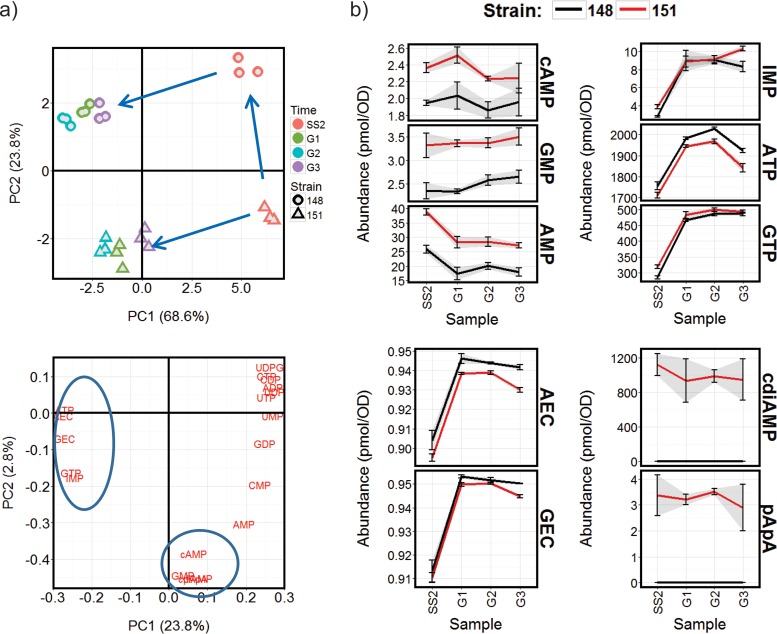
Operation of the ATP-cycling pathway constitutively reduces ATP abundance and AEC throughout the transition from steady-state growth on acetate to growth on glucose. (a) PCA of normalized nucleotide abundances (scores and loadings plots) identify the changes taking place that distinguish the induced ATP-cycling chemostats and the glucose-supplemented conditions, which are illustrated in detail in panel b. All nucleotide abundance profiles are available in Data Set S2.

**FIG 7:**
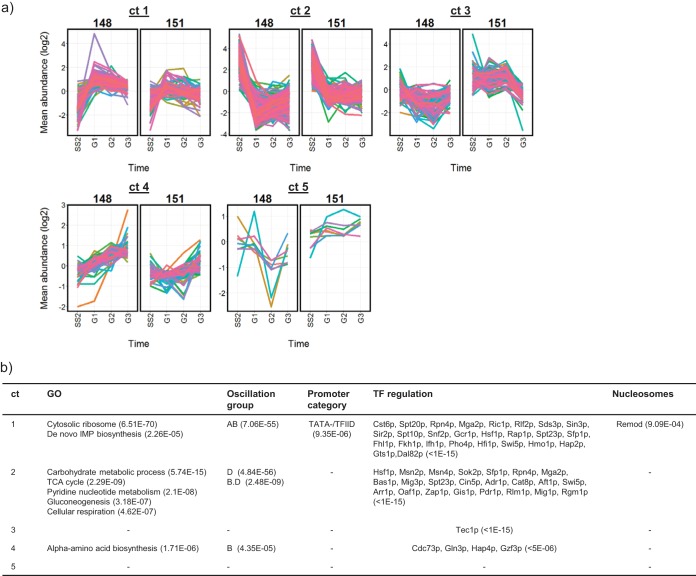
Induction of the ATP-cycling pathway partially inhibits the glucose repression of transcription following the addition of glucose to cultures growing at steady state on acetate. (a) Transcripts identified as being significantly differently expressed between chemostats of the control strain BY4741-112-148 (148) and the ATP-cycling strain BY4741-112-151 (151) were clustered, and (b) the clusters were functionally characterized as described in the legend to Fig. 3c. Data Set S4 provides full details of cluster membership and the enrichment analysis results.

Glucose is the preferred carbon source for yeast growth and, when provided to cells growing exclusively on a nonfermentable carbon source such as acetate, rapidly causes repression of the transcription of genes required for catabolism of the less favorable energy sources, and induces the transcription of genes required for growth (1, 8). The changes in gene transcription observed for clusters 2/4 and 1 in Fig. 3, respectively, are consistent with this change in carbon and energy metabolism. In the acetate-limited chemostats, comparison of the effects of glucose addition on gene transcription between the control and NTP-cycling strains identified 616 transcripts which behaved significantly differently in the ATP-cycling conditions, but only 13 during GTP cycling (Data Set S4). Both ATP abundance and AEC were consistently lower in the ATP-cycling cultures than in the corresponding control strain during the period following glucose addition, while cAMP, GMP, AMP, pApA, and cdiAMP were higher (Fig. 6). This coincided with a reduction in the extent of repression of a cluster of genes enriched for functions associated with carbon metabolism (cluster 2 in Fig. 7), and also with a decrease in the upregulation of genes enriched for functions associated with cell growth and proliferation (cluster 1 in Fig. 7). In the GTP-cycling chemostat cultures, only the GMP, pGpG, and cdiGMP nucleotides exhibited notable differences in abundance, and their glucose-dependent transcriptional reprogramming was unaffected (Fig. S3).

### Induction of the futile consumption of GTP produces only limited changes in the transcriptome.

Induction of the GTP-cycling pathway generated markedly fewer changes in the transcriptome than induction of ATP cycling in both the glucose and acetate chemostats: 77 transcripts were identified as being significantly changed in abundance relative to the control when grown on glucose, and only 53 during growth on acetate (Fig. S4 and S5 and Data Set S4). Both AEC and GEC remained unaffected by induction in either carbon condition; however, during the progression from SS1 to SS2 in the glucose-limited chemostats, ATP and GTP concentrations both fell, while that of CTP increased.

## DISCUSSION

**FIG 8:**
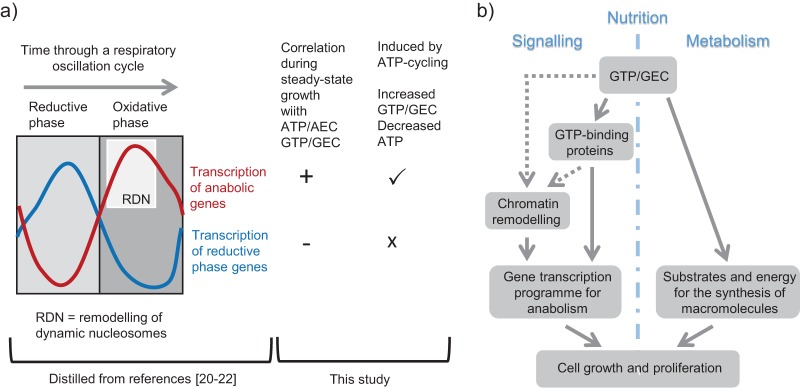
High-energy guanine nucleotides as a signal capable of linking growth to cellular energy status via the control of gene transcription. (a) Summary of the data indicating coordination of anabolic gene transcription with GTP/GEC. (b) GTP as a hub signaling molecule whose fluctuations in abundance depend on the interplay between nutrient supply and the rate of protein synthesis. GTP-binding proteins (regulatory GTPases or components of the actin cytoskeleton) are candidates for the observed modulation of gene transcription.

The effects of induction of the ATP-cycling pathway in this study, viewed in the context of the correlations observed between changes in nucleotide levels and the transcriptional programs occurring in cells growing in the control chemostats, support the proposal that yeast gene transcription is responsive to cell energy status (Fig. 8). Our data indicate, for the first time, a significant role for GTP and/or GEC in the signaling process. Since peptide bond formation during protein synthesis predominantly requires GTP, rather than ATP, this suggests a way for the cell to effect a direct linkage between nutritional status and the rate and extent of protein synthesis. This places GTP as a hub molecule whose fluctuations in abundance depend on the interplay between nutrient supply and the rate of protein synthesis and influence the regulation of gene expression (Fig. 8b).

Three main observations are indicative of the coordination of gene expression by changes in intracellular nucleotide use and composition. First, during continuous growth in chemostats, the transcription of genes required for glucose-dependent yeast cell proliferation was highly correlated with nucleotide indicators of high-energy status such as ATP, GTP, and IMP (Fig. 3 and 8). Second, the transcription of genes with functions associated with anabolic growth processes was upregulated following induction of the ATP-cycling pathway during growth on glucose and showed similarity to sets of genes previously identified as being associated with the oxidative growth phase of a respiratory oscillation cycle (Fig. 5 and 8). Finally, the reprogramming of gene expression that immediately follows release of cultures from dependence on the use of a less favorable nonfermentable carbon source (acetate) by the addition of glucose was partially inhibited during induction of ATP cycling (Fig. 7). This included reductions in the extent of both carbon catabolite repression and induction of ribosome biogenesis. Interestingly, induction of the GTP-cycling pathway under the same conditions could not reproduce these effects, and the consequences of inducing the ATP pathway during steady-state growth on acetate were notably different from those observed on glucose. Transcription of the bacterial pathway genes is comparable in each case (Fig. S1), but we cannot exclude posttranscriptional effects that may lead to differing fluxes through each engineered pathway. The design of the expression systems—where the bacterial synthetase and hydrolase enzymes are carried together on a plasmid that contains no internal regions of homology and whose selection depends on two auxotrophic markers (Fig. 1)—is expected to preclude the generation of subpopulations of cells that have lost either the plasmid or one or both of the bacterial genes. Such events are unlikely to generate subpopulations of any significant size over the moderate number of postinduction cell doublings used in the chemostat experiments (approximately six); however, this has not been formally assessed.

The ATP cycling-dependent changes in gene expression observed during steady-state growth on glucose are associated with increased GTP and GEC following induction. Changes in GTP, GEC, or cdiAMP were found to correlate most consistently with the changes in gene transcription, and we exclude cdiAMP, since a previous study revealed minimal effects on yeast gene transcription (36). Induction of intracellular concentrations of cdiAMP more than 5 times higher than the maximum observed in this work significantly changed the expression of only 75 S. cerevisiae genes during batch growth in YNB minimal medium containing 0.5% glucose (36). An important role for GEC and GTP in signaling metabolic status in S. cerevisiae has previously been proposed from an analysis of carbon and energy starvation in recombinant strains of S. cerevisiae engineered to ferment xylose (37). A decrease in guanine nucleotides is also a key signal for the initiation of meiosis and sporulation in yeast (38), and imbalances in guanine nucleotide pools are known to adversely affect cell metabolism and viability (39–41). Systems for sensing and responding to changes in intracellular guanine nucleotide composition therefore clearly exist, and this study provides evidence that they also participate in adjusting gene transcription to cellular energy status. The genes that are the target of this regulation under the experimental conditions used here tend to be those previously identified as possessing remodellable promoters and to be regulated by the SWI/SNF chromatin remodelling complex, suggesting that one of the signal outputs may be to promote the clearing of nucleosomes from susceptible promoters (21). Our data do not preclude a regulatory role for ATP and AEC sensing. Indeed the changes in gene transcription identified during the glucose derepression experiment more closely correlate with these nucleotides (discussed below). This observation of differing effects under different growth conditions suggests a network of regulation linking high-energy purine nucleotide abundance to gene transcription.

The influence of guanine nucleotide status on gene transcription could potentially be mediated by changes in the activation state of GTPases involved in the regulation of transcription in response to nutrient supply, for example Ras1p/Ras2p or Gtr1p/Gtr2p (1, 2, 6, 13). Evidence for an influence of guanine nucleotide pools on the level of active, GTP-bound, Ras2p has previously been reported (13, 15–17), and activation of the Ras2/cAMP/PKA pathway could produce the increase in expression of genes involved in ribosome biogenesis observed in the glucose chemostats in this study (Fig. 5).

Another possible route of control could be the balance between GTP and ATP inside the cells and its influence on transcription start site selection by RNA polymerase. One of the clusters of transcripts upregulated in the glucose-grown ATP-cycling chemostat cultures is significantly enriched for gene products which bind purine nucleotide triphosphates (cluster 3 in Fig. 5 and Data Set S4), raising the possibility that transcription of the genes encoding these binding proteins could in some way be sensitive to the prevailing levels of GTP (and/or ATP) in the cell. Interestingly, *de novo* purine biosynthesis is also upregulated under these conditions consistent with previous observations supporting transcription on demand for the genes in this pathway, controlled via sensing of key metabolic intermediates in the *de novo* pathway (42).

The eukaryotic cell cytoskeleton has also been proposed as an integrative sensor of metabolic status, capable of responding to, and influencing, intracellular concentrations of ATP and GTP through its use of these nucleotides as the energy source for polymerization and through its physical association with numerous metabolic enzymes, the translational apparatus, and mitochondria (43–45). While this is currently only an intriguing hypothesis, a cytoskeletal influence on gene transcription mediated through interactions with upstream kinases and transcription factors can also be imagined.

The reduction in glucose repression of gene transcription while the ATP-cycling pathway is fully operative coincides with increased intracellular concentrations of AMP, GMP, and cAMP (and cdiAMP) and with reduced ATP and AEC relative to the control strain (Fig. 7). This attenuation of the repression response under suboptimal energy conditions could make physiological sense, and it suggests a net inhibition of the activity of the Snf1 and Snf3/Rgt2 pathways which are central to the repression of gene transcription by glucose in *S. cerevisiae* (1, 8). However, Snf1p kinase activity is proposed to be modulated by ADP, not AMP or ATP, and increased activity of the Mig1p carbon-responsive repressor is predicted to result from less favorable energetic conditions. How this could be achieved is, therefore, not clear, although it is worth noting that the details of the *in vivo* regulation of Snf1p by adenine nucleotides remain to be fully established. Interestingly, *ACS1*, encoding an acetyl-CoA synthetase isoform expressed preferentially during growth on nonfermentable carbon sources (46), is repressed to only ca. 60% of the extent seen in the control strain (Fig. S6). This would be predicted to have consequences for nuclear acetyl-CoA abundance, with possible downstream effects on nucleosome remodelling and gene expression. Acetyl-CoA has been proposed as a sentinel metabolite capable of influencing chromatin structure, where increased nucleocytoplasmic concentrations increase the level of histone acetylation which in turn promotes nucleosome clearance and expression from susceptible promoter sequences (9, 23). The induction in transcription of growth-related genes in response to glucose addition is also dampened specifically during ATP cycling (Fig. 7). This suggests a reduced signaling through the TORC1 and PKA kinase pathways relative to the control strain under these conditions and implies an influence of nucleotide pools on these pathways.

10.1128/mBio.02500-18.6 FIG S6 Transcription of genes encoding the two acetyl-CoA synthetase enzymes in S. cerevisiae (ACS1 = TCONS_00000101; ACS2 = TCONS_00006154). Mean values ± standard deviations from triplicate chemostats are shown. Download FIG S6, EPS file, 1.9 MB. Copyright © 2019 Hesketh et al. 2019 Hesketh et al. This content is distributed under the terms of the Creative Commons Attribution 4.0 International license.

We conclude that a fundamental understanding of the global regulation of eukaryotic gene transcription will require a more detailed consideration of guanine nucleotide abundance and how this is signaled to the genome.

## MATERIALS AND METHODS

### Plasmid constructs and yeast strains.

The synthesis of cyclic diguanine monophosphate (cdiGMP) or cdiAMP in Saccharomyces cerevisiae was achieved using the engineered constructs previously described (36). For cyclic dinucleotide hydrolysis, synthetic genes designed with yeast codon usage to encode amino acids 395 to 649 of the cdiGMP hydrolase SFV3559 from Shigella flexneri (31) or amino acids 117 to 659 of the cdiAMP hydrolase YybT from Bacillus subtilis (32) were cloned into vectors between promoter and terminator sequences of the yeast gene *TDH3*. The truncated YybT enzyme lacks the two transmembrane helices present at the N terminus of the wild-type protein, and the C-terminal portion of SVF3559 contains the EAL domain phosphodiesterase active site. The TetO_7_pr::synthetase (with the VP16::TetR' TetO_7_ transactivator sequence) and TDH3pr::hydrolase expression constructs were finally combined as required in a centromeric vector pRS-LEU2-URA3 doubly marked with yeast *LEU2* and *URA3* genes (36) to produce the plasmids listed in Table 1 and illustrated in Fig. 1. All centromeric plasmids were transformed into the yeast laboratory strain BY4741 carrying an integrated copy of the SSN6::TetR fusion construct from pCM242 (26) on plasmid pAH112 (36).

### Verifying the activity of engineered strains by DOX induction in batch culture.

Flasks (250 ml) of YNB minimal medium (0.67% [wt/vol] yeast nitrogen base (Sigma) and 0.5% [wt/vol] ammonium sulfate) containing 2% glucose and 5 μg/ml doxycycline (DOX) were inoculated to a starting optical density at 600 nm (OD_600_) of 0.05 from overnight cultures. The inoculated cultures were incubated at 30°C with shaking at 200 rpm until reaching an OD_600_ of 0.4 to 0.5 before sampling for intracellular nucleotide extraction. Extracts were analyzed using HPLC-UV.

### Analyzing the effect of inducing increases in ATP or GTP consumption during steady-state growth in carbon-limited chemostat cultures.

Chemostat fermentations were performed in triplicate under carbon-limited conditions in minimal media using either glucose or acetate as the carbon source and providing only ammonium sulfate and essential trace nutrients as supplements. For cultures in glucose-grown chemostats, precultures of each strain were prepared by inoculating YNB minimal medium (0.67% [wt/vol] yeast nitrogen base [Sigma] and 0.5% [wt/vol] ammonium sulfate) containing 2% (wt/vol) glucose with a single colony picked from an agar plate and incubating at 30°C 200 rpm for 24 h. Fermentors (2 liters) containing F1 medium (47) (1,000 ml) with 0.25% (wt/vol) glucose were inoculated with aliquots of the precultures to produce a starting OD_600_ of 0.05 and grown in batch for 24 h (30°C, 750 rpm stirrer speed, aeration with 1 liter min^−1^ air). Cultures were then switched to continuous mode, maintaining the pH at 4.5 and the dilution rate at 0.11 to 0.12 h^−1^. Biomass was monitored offline at regular intervals by measuring UV absorbance at a wavelength of 600 nm, and the purity of the cultures was routinely checked by phase-contrast microscopy. Initial steady-state culture conditions (designated SS1) were deemed to have been established after more than five vessel volume changes and with cultures exhibiting a constant biomass at which point, culture samples were taken for analysis. For induction, DOX was added to the steady-state cultures to produce a final concentration of 5 μg/ml. Culture samples were harvested for analysis at 3, 6, and 9 h after induction, and a final sample taken after the cultures had reached a new steady state (SS2), as determined by the passage of six vessel volume changes and with cultures exhibiting a constant biomass.

A similar protocol was followed for the acetate-grown chemostat experiments, except the initial preculture was performed for 42 h in YNB minimal medium containing 0.5% (wt/vol) sodium acetate and 1% (wt/vol) glucose. This was used to inoculate batch growth in fermentors containing YNB minimal medium with 0.5% (wt/vol) sodium acetate and 0.05% (wt/vol) glucose and then switched to continuous mode using only 0.5% (wt/vol) sodium acetate as the carbon source and controlling the pH at 5.0 and the dilution rate at 0.07 to 0.08 h^−1^. YNB was used in preference to F1 medium, since strains grew less well in the latter when only acetate was present as the carbon source. After taking the induced steady-state samples, designated SS2, an additional experiment was performed to analyze the response to adding glucose to the respiring, induced cultures. Glucose (40% [wt/vol]) was added to each fermentor (and to the feed for each fermentor) to produce a final concentration of 1.6% (wt/vol), and culture samples were taken for RNA and intracellular nucleotide extraction at 15 min (designated G1), 30 min (designated G2), and 60 min (designated G3) after glucose addition. All nucleotide extracts from the chemostat experiments were analyzed using the LC-MS method.

### Preparation of intracellular nucleotide extracts.

Cell metabolism in culture samples was immediately quenched by transferring culture aliquots (10 ml) directly to methanol (40 ml) cooled to below −60°C on dry ice and standing for 2 min. Cells from quenched samples were harvested by centrifugation at −20°C and extracted by resuspension in ice-cold 1 N formic acid containing 10% butan-1-ol, standing on ice for 60 min. Cells were removed from extracts by centrifugation at −20°C, and the supernatants were filtered through a 0.45-μm filter and stored frozen at −80°C. Frozen extracts were evaporated to dryness by freeze-drying and finally reconstituted in 200 μl water.

### Analysis of intracellular nucleotides by HPLC-UV.

Separation and quantification of intracellular nucleotides by HPLC-UV were performed essentially by the method of Strauch et al. (48) using an Agilent HP1100 HPLC. Briefly, aliquots of nucleotide extracts were separated at 20°C by anion-exchange chromatography using a Partisil 10SAX column (10-μm particle size; 25 cm by 4.6 mm internal diameter). Gradient elution was performed by the following gradient elution program using mobile phase A (MPA) (7 mM potassium hydrogen phosphate [pH 4.0]) and mobile phase B (MPB) (0.5 M potassium dihydrogen phosphate–0.5 M sodium sulfate pH 5.4) and a flow rate of 1.5 ml/min: 0 to 5 min, 100% MPA; 5 to 10 min, 100% to 85% MPA; 10 to 15 min, 85% to 81% MPA; 15 to 20 min, 81% to 50% MPA; 20 to 25 min, 50% to 30% MPA; 25 to 30 min, 30% to 25% MPA; 30 to 40 min, 30% to 0% MPA (postrun equilibration, 100% MPA for 15 min). Eluting nucleotides were detected using a UV diode-array detector and quantified by their absorbance at 254 nm in comparison to known standards. Quantified values in picomoles were normalized to the cells present in 1 ml of a culture with an OD_600_ value of 1. All nucleotide standards were purchased from Sigma, with the exception of cdiAMP, cdiGMP, pApA, and pGpG which came from Biolog.

### Analysis of intracellular nucleotides by LC-MS.

Separation and quantification of intracellular nucleotides by LC-MS were performed using an Agilent HP1290 LC system attached to an Agilent 6460 triple quad mass spectrometer by a method based on one devised for the analysis of thiopurine nucleotides (49). Briefly, aliquots of nucleotide extracts were separated at 30°C by anion-exchange chromatography using a Biobasic AX column (5-μm particle size; 5 cm by 2.1 mm internal diameter) and the following gradient elution program using mobile phase A (MPA) (10 mM ammonium acetate in water-acetonitrile [7:3] [pH 6]) and mobile phase B (MPB) (1 mM ammonium acetate in water-acetonitrile [7:3] [pH 10.5]): 0% to 100% MPB in 5.75 min (postrun equilibration 100% MPA for 4.25 min) at a flow rate of 0.5 ml/min. Nucleotides were detected in the mass spectrometer using the multiple reaction monitoring (MRM) mode with the following settings: capillary voltage, 3,500 V; nozzle voltage, 1,000 V; drying gas flow, 10 liters/min; drying gas temperature, 325°C; nebulizer pressure, 10 lb/in^2^; sheath gas flow, 11 liters/min; sheath gas temperature, 350°C. Nitrogen was used as the sheath, nebulizer, drying, and collision gases. Quantification was performed using the Agilent MassHunter software using calibration curves generated from the contemporary analysis of known standards. Quantified values in picomoles were normalized to the value for cells present in 1 ml of a culture with an OD_600_ value of 1 and then processed to generate the same total value of common nucleotides in each sample for each chemostat condition (growth in glucose or acetate). The following MRM mass transitions (listed as parent ion mass to fragment ion mass) were used for detection and identification of the nucleotides in the positive ion mode (except for UDP-glucose, which was detected in the negative ion mode): ATP, 508.1 to 136.0; ADP, 428.2 to 136.0; AMP, 348.2 to 136.0; cAMP, 330.2 to 136.0; cdiAMP, 659.4 to 136.1; pApA, 677.5 to 136.1; GTP, 524.2 to 152.0; GDP, 444.2 to 152.0; GMP, 364.2 to 152.0; cdiGMP, 691.4 to 152.2; pGpG, 709.4 to 152.2; IMP, 349.2 to 157.0; UTP, 485.1 to 97.0; UDP, 405.1 to 97.0; UMP, 325.1 to 97.0; UDP-glucose, 565.3 to 323.0; CTP, 484.1 to 112.0; CDP, 404.2 to 112.0; CMP, 325.1 to 112.0.

### Isolation of total RNA.

Cells from culture samples were harvested rapidly by centrifugation, and the cell pellets were flash frozen in liquid nitrogen and stored at −80°C until required. For RNA purification, frozen cells were resuspended in TRIzol (Invitrogen) and lysed mechanically at 4°C by bead beating using a FastPrep homogenizer (MP Biomedicals; five 1-min cycles of shaking at 6 m/s). DNA and protein were removed from the lysed samples by extraction with chloroform (twice), and total RNA was purified using RNeasy columns (Qiagen).

### Transcriptomics analysis using RNAseq.

Strand-specific transcriptome sequencing was performed by a commercial sequencing provider using an Illumina HiSeq sequencer. Poly(A)-containing mRNA molecules were purified and sequenced to provide a minimum of 15 million 50-bp single-end reads per sample. Sequencing reads were mapped to the S. cerevisiae S288C genome sequence (modified to include the heterologous genes introduced) using tophat v2.0.14 (50), employing the default settings with the following exceptions: min-intron length = 25; max-intron length = 1,500. A universal set of transcripts was assembled from all the data using stringTie (51), combining the result with the reference genome annotation S288C_reference_genome_R64-2-1_20150113 (http://www.yeastgenome.org/). Mapped reads were processed and analyzed in R (52). Reads mapping to features were counted using Rsubread (53), and the transcript counts were TMM normalized and tested for differential expression using the limma voom method (54, 55) with limma treat (56). Gene ontology (GO) analysis was performed using goseq (57), and principal components analysis was realized using pcaMethods (58). To identify sets of differentially expressed transcripts in each chemostat experiment, the null hypothesis that the change in transcript abundance relative to the preinduced/pretreated time point was equal in each control and NTP-cycling strain pair was assumed, e.g., [NTP.SS2 − NTP.SS1] − [Ctrl.SS2 − Ctrl.SS1] = 0 or [NTP.G3 − NTP.SS2] − [Ctrl.G3 − Ctrl.SS2] = 0. A limma treat transcript fold change threshold of 1.25 was used. The analysis was performed for each postinduction/posttreatment time point, and the transcripts identified as being significantly (*q* ≤ 0.05) differently expressed in each experiment were combined. The abundance profiles of significant transcripts were clustered, and each cluster was subjected to GO analysis, regulatory interaction enrichment analysis using yeastract (35), and enrichment analysis for membership of functionally associated groups obtained from the literature using Fisher’s exact test.

### Other data analysis.

Correlations between nucleotide and transcript abundance data were analyzed using the mixOmics R package (mixOmics: Omics data integration project [http://mixomics.org/]). Nucleotide abundances were log_2_ transformed prior to correlation with normalized RPKM transcript abundance values obtained from the limma voom normalized count data. Pearson correlations were calculated using “correlation” and “ward” as the distance and clustering settings, respectively, and applying the threshold values stated in the text (minimum, 0.7).

### Accession number(s).

RNAseq data are available in the ArrayExpress database (http://www.ebi.ac.uk/arrayexpress) under accession number E-MTAB-5174.

## References

[1] Broach J 2012 Nutritional control of growth and development in yeast. Genetics 192:73–105. doi:10.1534/genetics.111.135731. 22964838 PMC3430547

[2] Conrad M, Schothorst J, Kankipati HN, Van Zeebroeck G, Rubio-Texeira M, Thevelein JM 2014 Nutrient sensing and signaling in the yeast Saccharomyces cerevisiae. FEMS Microbiol Rev 38:254–299. doi:10.1111/1574-6976.12065. 24483210 PMC4238866

[3] Hardie DG, Schaffer BE, Brunet A 2016 AMPK: an energy-sensing pathway with multiple inputs and outputs. Trends Cell Biol 26:190–201. doi:10.1016/j.tcb.2015.10.013. 26616193 PMC5881568

[4] Loewith R, Hall M 2011 Target of rapamycin (TOR) in nutrient signaling and growth control. Genetics 189:1177–1201. doi:10.1534/genetics.111.133363. 22174183 PMC3241408

[5] Smets B, Ghillebert R, De Snijder P, Binda M, Swinnen E, De Virgilio C, Winderickx J 2010 Life in the midst of scarcity: adaptations to nutrient availability in Saccharomyces cerevisiae. Curr Genet 56:1–32. doi:10.1007/s00294-009-0287-1. 20054690

[6] Péli-Gulli M-PP, Sardu A, Panchaud N, Raucci S, De Virgilio C 2015 Amino acids stimulate TORC1 through Lst4-Lst7, a GTPase-activating protein complex for the Rag family GTPase Gtr2. Cell Rep 13:1–7. doi:10.1016/j.celrep.2015.08.059. 26387955

[7] Toda T, Cameron S, Sass P, Zoller M, Wigler M 1987 Three different genes in S. cerevisiae encode the catalytic subunits of the cAMP-dependent protein kinase. Cell 50:277–287. doi:10.1016/0092-8674(87)90223-6. 3036373

[8] Busti S, Coccetti P, Alberghina L, Vanoni M 2010 Glucose signaling-mediated coordination of cell growth and cell cycle in Saccharomyces cerevisiae. Sensors (Basel) 10:6195–6240. doi:10.3390/s100606195. 22219709 PMC3247754

[9] Cai L, Sutter BM, Li B, Tu BP 2011 Acetyl-CoA induces cell growth and proliferation by promoting the acetylation of histones at growth genes. Mol Cell 42:426–437. doi:10.1016/j.molcel.2011.05.004. 21596309 PMC3109073

[10] Mayer FV, Heath R, Underwood E, Sanders MJ, Carmena D, McCartney RR, Leiper FC, Xiao B, Jing C, Walker PA, Haire LF, Ogrodowicz R, Martin SR, Schmidt MC, Gamblin SJ, Carling D 2011 ADP regulates SNF1, the Saccharomyces cerevisiae homolog of AMP-activated protein kinase. Cell Metabolism 14:707–714. doi:10.1016/j.cmet.2011.09.009. 22019086 PMC3241989

[11] Atkinson DE, Walton GM 1967 Adenosine triphosphate conservation in metabolic regulation. Rat liver citrate cleavage enzyme. J Biol Chem 242:3239–3241. 6027798

[12] Thompson FM, Atkinson DE 1971 Response of nucleoside diphosphate kinase to the adenylate energy charge. Biochem Biophys Res Commun 45:1581–1585. doi:10.1016/0006-291X(71)90201-4. 5166850

[13] Rudoni S, Colombo S, Coccetti P, Martegani E 2001 Role of guanine nucleotides in the regulation of the Ras/cAMP pathway in Saccharomyces cerevisiae. Biochim Biophys Acta 1538:181–189. doi:10.1016/S0167-4889(01)00067-2. 11336789

[14] Walther T, Novo M, Rössger K, Létisse F, Loret MO, Portais JC, François JM 2010 Control of ATP homeostasis during the respiro-fermentative transition in yeast. Mol Syst Biol 6:344. doi:10.1038/msb.2009.100. 20087341 PMC2824524

[15] Pescini D, Cazzaniga P, Besozzi D, Mauri G, Amigoni L, Colombo S, Martegani E 2012 Simulation of the Ras/cAMP/PKA pathway in budding yeast highlights the establishment of stable oscillatory states. Biotechnol Adv 30:99–107. doi:10.1016/j.biotechadv.2011.06.014. 21741466

[16] Besozzi D, Cazzaniga P, Pescini D, Mauri G, Colombo S, Martegani E 2012 The role of feedback control mechanisms on the establishment of oscillatory regimes in the Ras/cAMP/PKA pathway in S. cerevisiae. EURASIP J Bioinform Syst Biol 2012:10. doi:10.1186/1687-4153-2012-10. 22818197 PMC3479052

[17] Cazzaniga P, Pescini D, Besozzi D, Mauri G, Colombo S, Martegani E 2008 Modeling and stochastic simulation of the Ras/cAMP/PKA pathway in the yeast Saccharomyces cerevisiae evidences a key regulatory function for intracellular guanine nucleotides pools. J Biotechnol 133:377–385. doi:10.1016/j.jbiotec.2007.09.019. 18023904

[18] Klevecz RR, Bolen J, Forrest G, Murray DB 2004 A genomewide oscillation in transcription gates DNA replication and cell cycle. Proc Natl Acad Sci U S A 101:1200–1205. doi:10.1073/pnas.0306490101. 14734811 PMC337030

[19] Tu BP, Kudlicki A, Rowicka M, McKnight SL 2005 Logic of the yeast metabolic cycle: temporal compartmentalization of cellular processes. Science 310:1152–1158. doi:10.1126/science.1120499. 16254148

[20] Amariei C, Machné R, Sasidharan K, Gottstein W, Tomita M, Soga T 2013 The dynamics of cellular energetics during continuous yeast culture. Conf Proc IEEE Eng Med Biol Soc 2013:2708–2711. doi:10.1109/EMBC.2013.6610099. 24110286

[21] Nocetti N, Whitehouse I 2016 Nucleosome repositioning underlies dynamic gene expression. Genes Dev 30:660–672. doi:10.1101/gad.274910.115. 26966245 PMC4803052

[22] Machné R, Murray DB 2012 The yin and yang of yeast transcription: elements of a global feedback system between metabolism and chromatin. PLoS One 7:e37906. doi:10.1371/journal.pone.0037906. 22685547 PMC3369881

[23] Shi L, Tu BP 2014 Protein acetylation as a means to regulate protein function in tune with metabolic state. Biochem Soc Trans 42:1037–1042. doi:10.1042/BST20140135. 25109999

[24] Castrillo JI, Zeef LA, Hoyle DC, Zhang N, Hayes A, Gardner DC, Cornell MJ, Petty J, Hakes L, Wardleworth L, Rash B, Brown M, Dunn WB, Broadhurst D, O'Donoghue K, Hester SS, Dunkley TP, Hart SR, Swainston N, Li P, Gaskell SJ, Paton NW, Lilley KS, Kell DB, Oliver SG 2007 Growth control of the eukaryote cell: a systems biology study in yeast. J Biol 6:4. doi:10.1186/jbiol54. 17439666 PMC2373899

[25] Gutteridge A, Pir P, Castrillo JI, Charles PD, Lilley KS, Oliver SG 2010 Nutrient control of eukaryote cell growth: a systems biology study in yeast. BMC Biol 8:68. doi:10.1186/1741-7007-8-68. 20497545 PMC2895586

[26] Bellí G, Garí E, Piedrafita L, Aldea M, Herrero E 1998 An activator/repressor dual system allows tight tetracycline-regulated gene expression in budding yeast. Nucleic Acids Res 26:942–947. doi:10.1093/nar/26.4.942. 9461451 PMC147371

[27] Ludwig JR, Oliver SG, McLaughlin CS 1977 The effect of amino acids on growth and phosphate metabolism in a prototrophic yeast strain. Biochem Biophys Res Commun 79:16–23. doi:10.1016/0006-291X(77)90054-7. 336043

[28] Alam MT, Zelezniak A, Mülleder M, Shliaha P, Schwarz R, Capuano F, Vowinckel J, Radmaneshfar E, Krüger A, Calvani E, Michel S, Börno S, Christen S, Patil KR, Timmermann B, Lilley KS, Ralser M 2016 The metabolic background is a global player in Saccharomyces gene expression epistasis. Nat Microbiol 1:15030. doi:10.1038/nmicrobiol.2015.30. 27572163 PMC5131842

[29] Christen B, Christen M, Paul R, Schmid F, Folcher M, Jenoe P, Meuwly M, Jenal U 2006 Allosteric control of cyclic di-GMP signaling. J Biol Chem 281:32015–32024. doi:10.1074/jbc.M603589200. 16923812

[30] Mehne F, Gunka K, Eilers H, Herzberg C, Kaever V, Stülke J 2013 Cyclic di-AMP homeostasis in Bacillus subtilis: both lack and high-level accumulation of the nucleotide are detrimental to cell growth. J Biol Chem 288:2004–2017. doi:10.1074/jbc.M112.395491. 23192352 PMC3548507

[31] Tchigvintsev A, Xu X, Singer A, Chang C, Brown G, Proudfoot M, Cui H, Flick R, Anderson WF, Joachimiak A, Galperin MY, Savchenko A, Yakunin AF 2010 Structural insight into the mechanism of c-di-GMP hydrolysis by EAL domain phosphodiesterases. J Mol Biol 402:524–538. doi:10.1016/j.jmb.2010.07.050. 20691189 PMC2945410

[32] Rao F, See R, Zhang D, Toh D, Ji Q, Liang Z-X 2010 YybT is a signaling protein that contains a cyclic dinucleotide phosphodiesterase domain and a GGDEF domain with ATPase activity. J Biol Chem 285:473–482. doi:10.1074/jbc.M109.040238. 19901023 PMC2804195

[33] Rohart F, Gautier B, Singh A, Lê Cao K-AA 2017 mixOmics: an R package for ’omics feature selection and multiple data integration. PLoS Comput Biol 13:e1005752. doi:10.1371/journal.pcbi.1005752. 29099853 PMC5687754

[34] Rhee HS, Pugh BF 2012 Genome-wide structure and organization of eukaryotic pre-initiation complexes. Nature 483:295–301. doi:10.1038/nature10799. 22258509 PMC3306527

[35] Teixeira MC, Monteiro PT, Guerreiro JF, Gonçalves JP, Mira NP, dos Santos SC, Cabrito TR, Palma M, Costa C, Francisco AP, Madeira SC, Oliveira AL, Freitas AT, Sá-Correia I 2014 The YEASTRACT database: an upgraded information system for the analysis of gene and genomic transcription regulation in Saccharomyces cerevisiae. Nucleic Acids Res 42:D161–D166. doi:10.1093/nar/gkt1015. 24170807 PMC3965121

[36] Hesketh A, Vergnano M, Wan C, Oliver SG 2017 Bacterial signaling nucleotides inhibit yeast cell growth by impacting mitochondrial and other specifically eukaryotic functions. mBio 8:e01047-17. doi:10.1128/mBio.01047-17. 28743817 PMC5527313

[37] Bergdahl B, Heer D, Sauer U, Hahn-Hägerdal B, Niel E 2012 Dynamic metabolomics differentiates between carbon and energy starvation in recombinant Saccharomyces cerevisiae fermenting xylose. Biotechnol Biofuels 5:34. doi:10.1186/1754-6834-5-34. 22587303 PMC3462113

[38] Varma A, Freese EB, Freese E 1985 Partial deprivation of GTP initiates meiosis and sporulation in Saccharomyces cerevisiae. Mol Gen Genet 201:1–6. doi:10.1007/BF00397977. 3903431

[39] Iglesias-Gato D, Martín-Marcos P, Santos MAA, Hinnebusch AG, Tamame M 2011 Guanine nucleotide pool imbalance impairs multiple steps of protein synthesis and disrupts GCN4 translational control in Saccharomyces cerevisiae. Genetics 187:105–122. doi:10.1534/genetics.110.122135. 20980241 PMC3018310

[40] Saint-Marc C, Pinson B, Coulpier F, Jourdren L, Lisova O, Daignan-Fornier B 2009 Phenotypic consequences of purine nucleotide imbalance in Saccharomyces cerevisiae. Genetics 183:529–538. doi:10.1534/genetics.109.105858. 19635936 PMC2766314

[41] Breton A, Pinson B, Coulpier F, Giraud M-FF, Dautant A, Daignan-Fornier B 2008 Lethal accumulation of guanylic nucleotides in Saccharomyces cerevisiae HPT1-deregulated mutants. Genetics 178:815–824. doi:10.1534/genetics.107.083295. 18245832 PMC2248339

[42] Rolfes RJ 2006 Regulation of purine nucleotide biosynthesis: in yeast and beyond. Biochem Soc Trans 34:786–790. doi:10.1042/BST0340786. 17052198

[43] Norris V, Amar P, Legent G, Ripoll C, Thellier M, Ovádi J 2013 Sensor potency of the moonlighting enzyme-decorated cytoskeleton: the cytoskeleton as a metabolic sensor. BMC Biochem 14:3. doi:10.1186/1471-2091-14-3. 23398642 PMC3577492

[44] Sattlegger E, Chernova TA, Gogoi NM, Pillai IV, Chernoff YO, Munn AL 2014 Yeast studies reveal moonlighting functions of the ancient actin cytoskeleton. IUBMB Life 66:538–545. doi:10.1002/iub.1294. 25138357 PMC4176509

[45] Aon MA, Cortassa S 2014 Function of metabolic and organelle networks in crowded and organized media. Front Physiol 5:523. doi:10.3389/fphys.2014.00523. 25653618 PMC4300868

[46] de Jong-Gubbels P, van den Berg MA, Steensma HY, van Dijken JP, Pronk JT 2006 The Saccharomyces cerevisiae acetyl-coenzyme A synthetase encoded by the ACS1 gene, but not the ACS2-encoded enzyme, is subject to glucose catabolite inactivation. FEMS Microbiol Lett 153:75–81. doi:10.1111/j.1574-6968.1997.tb10466.x. 9252575

[47] Hayes A, Zhang N, Wu J, Butler PR, Hauser NC, Hoheisel JD, Lim FL, Sharrocks AD, Oliver SG 2002 Hybridization array technology coupled with chemostat culture: tools to interrogate gene expression in Saccharomyces cerevisiae. Methods 26:281–290. doi:10.1016/S1046-2023(02)00032-4. 12054884

[48] Strauch E, Takano E, Baylis HA, Bibb MJ 1991 The stringent response in Streptomyces coelicolor A3(2). Mol Microbiol 5:289–298. doi:10.1111/j.1365-2958.1991.tb02109.x. 1710311

[49] Hofmann U, Heinkele G, Angelberger S, Schaeffeler E, Lichtenberger C, Jaeger S, Reinisch W, Schwab M 2012 Simultaneous quantification of eleven thiopurine nucleotides by liquid chromatography-tandem mass spectrometry. Anal Chem 84:1294–1301. doi:10.1021/ac2031699. 22220820

[50] Kim D, Pertea G, Trapnell C, Pimentel H, Kelley R, Salzberg SL 2013 TopHat2: accurate alignment of transcriptomes in the presence of insertions, deletions and gene fusions. Genome Biol 14:R36. doi:10.1186/gb-2013-14-4-r36. 23618408 PMC4053844

[51] Pertea M, Pertea GM, Antonescu CM, Chang T-CC, Mendell JT, Salzberg SL 2015 StringTie enables improved reconstruction of a transcriptome from RNA-seq reads. Nat Biotechnol 33:290–295. doi:10.1038/nbt.3122. 25690850 PMC4643835

[52] R Core Team. 2015 R: a language and environment for statistical computing. R Foundation for Statistical Computing, Vienna, Austria.

[53] Liao Y, Smyth GK, Shi W 2013 The Subread aligner: fast, accurate and scalable read mapping by seed-and-vote. Nucleic Acids Res 41:e108. doi:10.1093/nar/gkt214. 23558742 PMC3664803

[54] Liu R, Holik AZ, Su S, Jansz N, Chen K, Leong HS, Blewitt ME, Asselin-Labat M-L, Smyth GK, Ritchie ME 2015 Why weight? Modelling sample and observational level variability improves power in RNA-seq analyses. Nucleic Acids Res 43:e97. doi:10.1093/nar/gkv412. 25925576 PMC4551905

[55] Law CW, Chen Y, Shi W, Smyth GK 2014 voom: precision weights unlock linear model analysis tools for RNA-seq read counts. Genome Biol 15:R29. doi:10.1186/gb-2014-15-2-r29. 24485249 PMC4053721

[56] McCarthy DJ, Smyth GK 2009 Testing significance relative to a fold-change threshold is a TREAT. Bioinformatics 25:765–771. doi:10.1093/bioinformatics/btp053. 19176553 PMC2654802

[57] Young MD, Wakefield MJ, Smyth GK, Oshlack A 2010 Gene ontology analysis for RNA-seq: accounting for selection bias. Genome Biol 11:R14. doi:10.1186/gb-2010-11-2-r14. 20132535 PMC2872874

[58] Stacklies W, Redestig H, Scholz M, Walther D, Selbig J 2007 pcaMethods–a bioconductor package providing PCA methods for incomplete data. Bioinformatics 23:1164–1167. doi:10.1093/bioinformatics/btm069. 17344241

