# Supplementary material for: Determination of the Global Pattern of Gene Expression in Yeast Cells by Intracellular Levels of Guanine Nucleotides
Source: mBio. 2019 Jan 22;10(1):e02500-18. doi: 10.1128/mBio.02500-18 (PMC6343037; doi:10.1128/mBio.02500-18)
Supplement: DATA SET S2 [file mBio.02500-18-sd002.pdf]

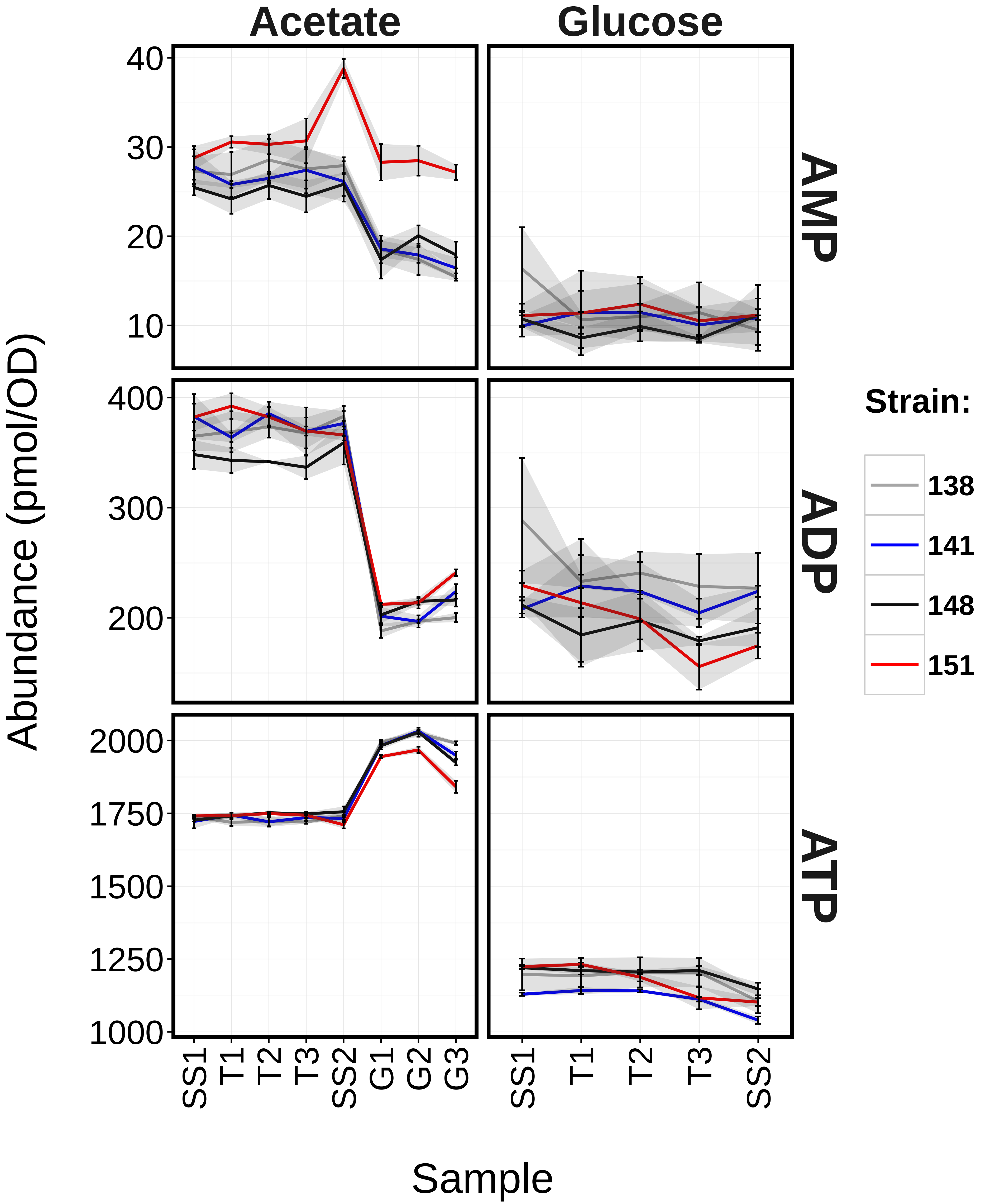
Acetate
Glucose
AMP
ADP
ATP
Abundance (pmol/OD)
Sample
Strain:
138
141
148
151
40
30
20
10
400
300
200
2000
1750
1500
1250
1000
SS1
T1
T2
T3
SS2
G1
G2
G3

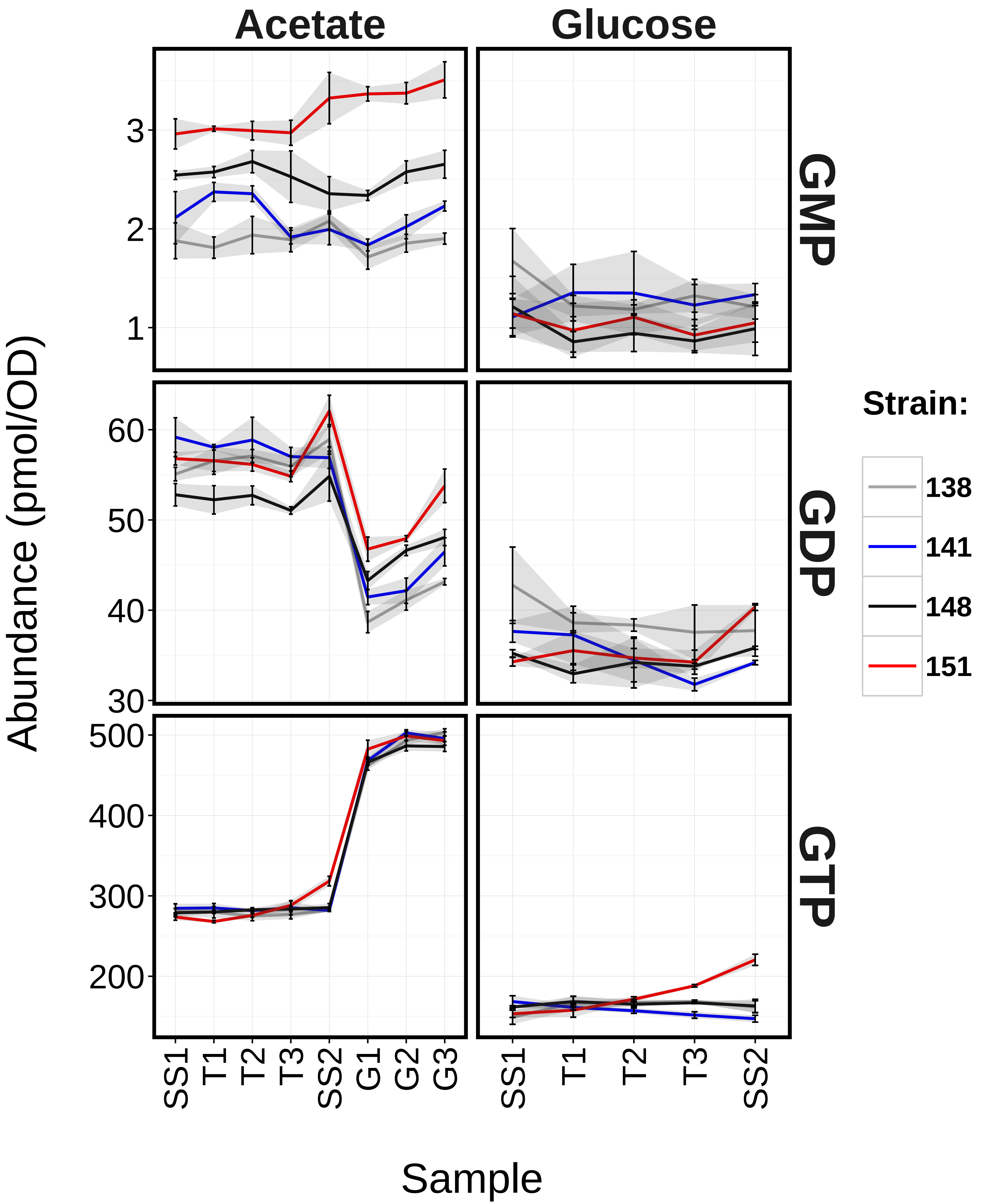

Acetate
Glucose
GMP
GDP
GTP
Abundance (pmol/OD)
Sample
3
2
1
60
50
40
30
500
400
300
200
SS1
T1
T2
T3
SS2
G1
G2
G3
Strain:
138
141
148
151

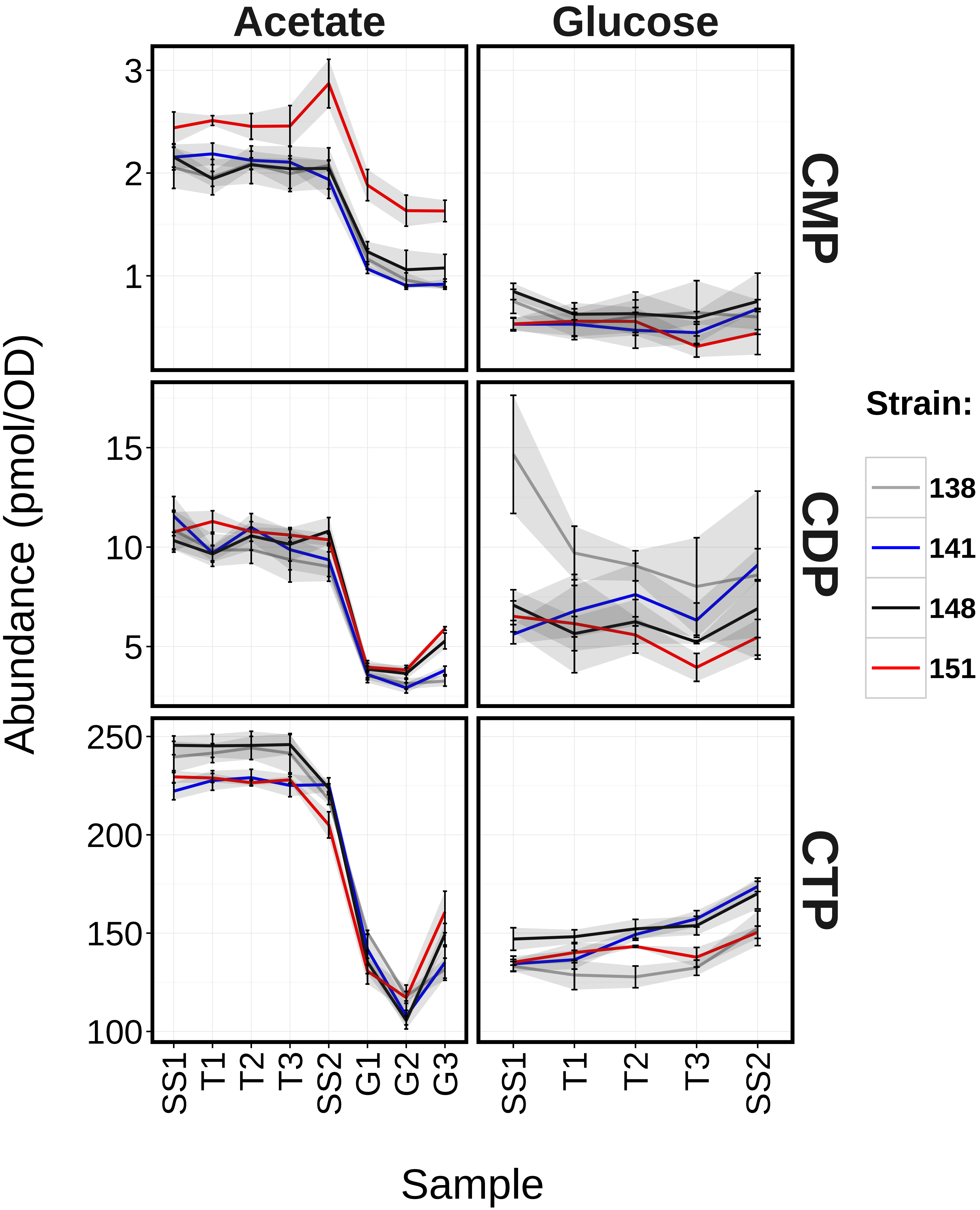

Acetate
Glucose
CMP
CDP
CTP
Abundance (pmol/OD)
Sample
Strain:
138
141
148
151
3
2
1
15
10
5
250
200
150
100
SS1
T1
T2
T3
SS2
G1
G2
G3

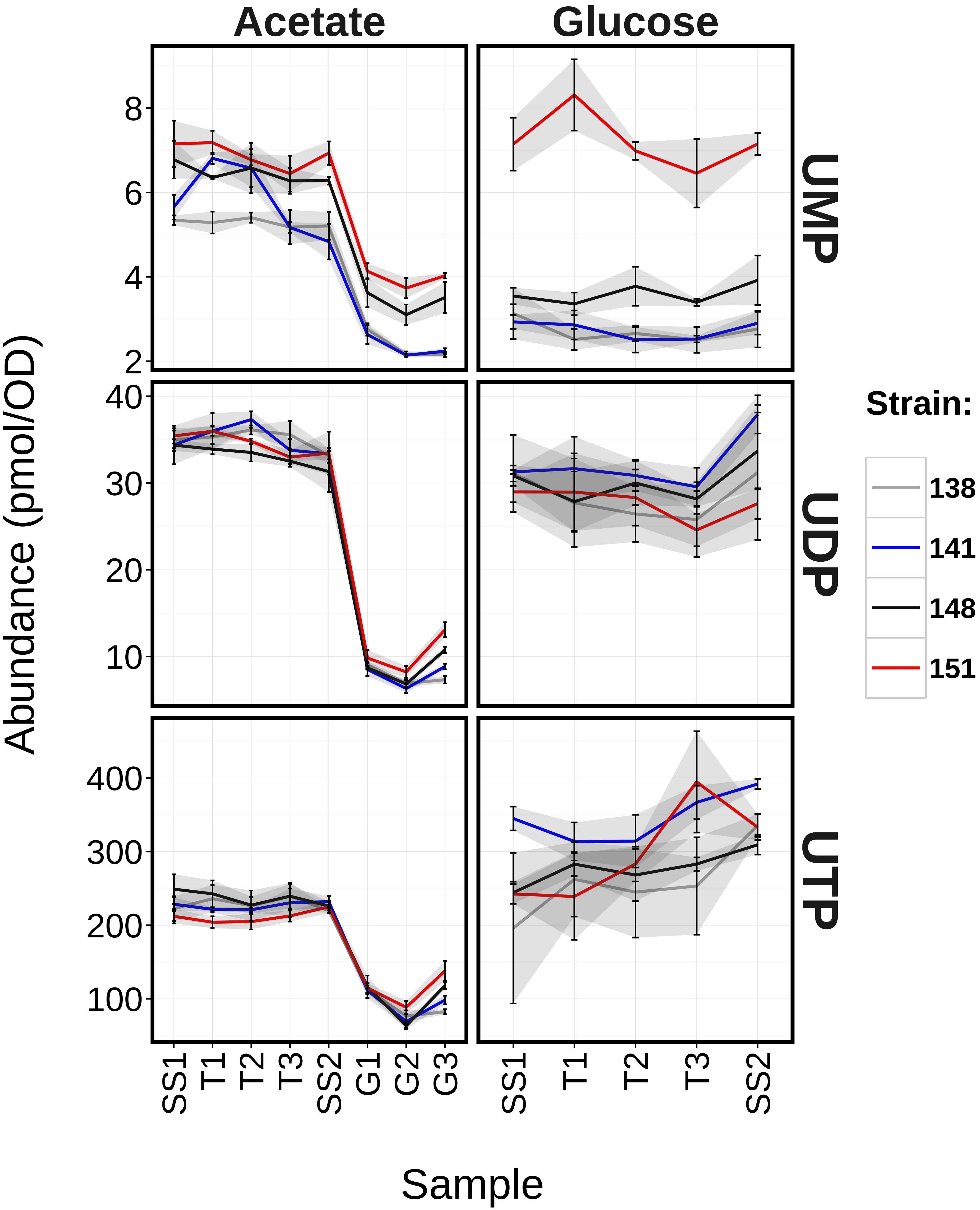
Acetate
Glucose
UMP
UDP
UTP
Abundance (pmol/OD)
Sample
8
6
4
2
40
30
20
10
400
300
200
100
SS1
T1
T2
T3
SS2
G1
G2
G3
Strain:
138
141
148
151

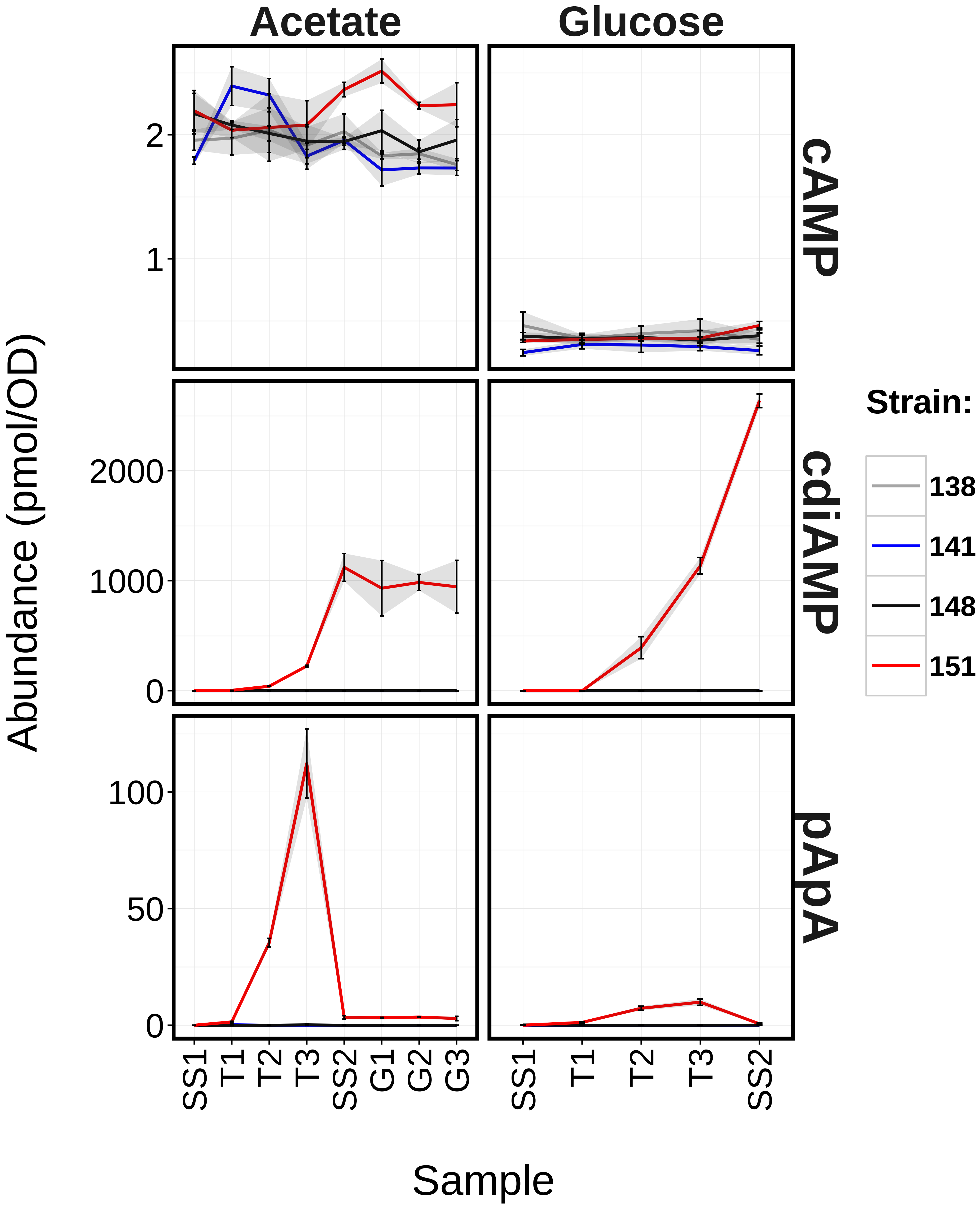

Acetate
Glucose
cAMP
cdiAMP
pApA
Abundance (pmol/OD)
Sample
Strain:
138
141
148
151
2
1
2000
1000
0
100
50
0
SS1
T1
T2
T3
SS2
G1
G2
G3
SS1
T1
T2
T3
SS2

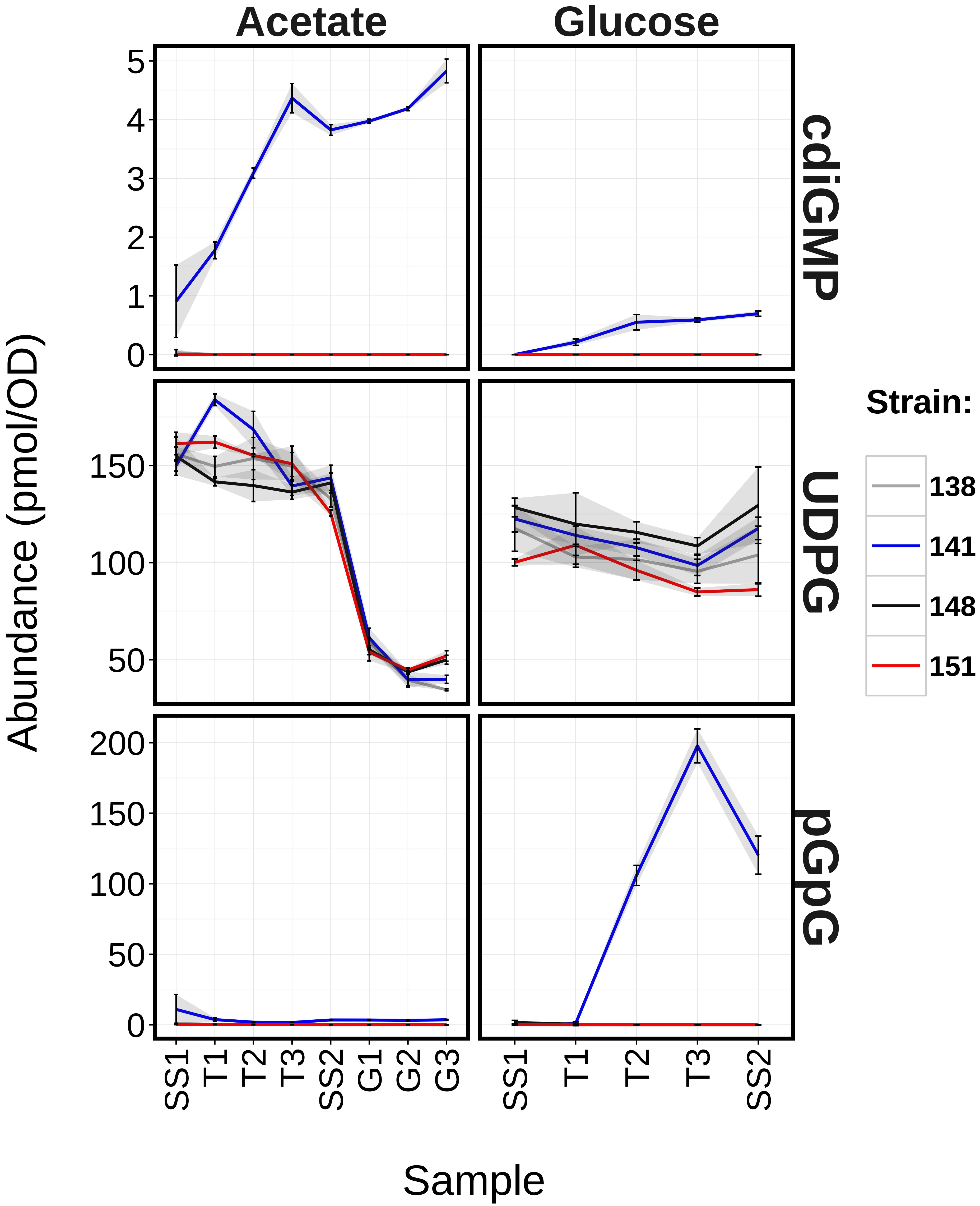

Acetate
Glucose
cdiGMP
UDPG
pGpG
Abundance (pmol/OD)
Sample
Strain:
138
141
148
151
0
1
2
3
4
5
50
100
150
200
SS1
T1
T2
T3
SS2
G1
G2
G3

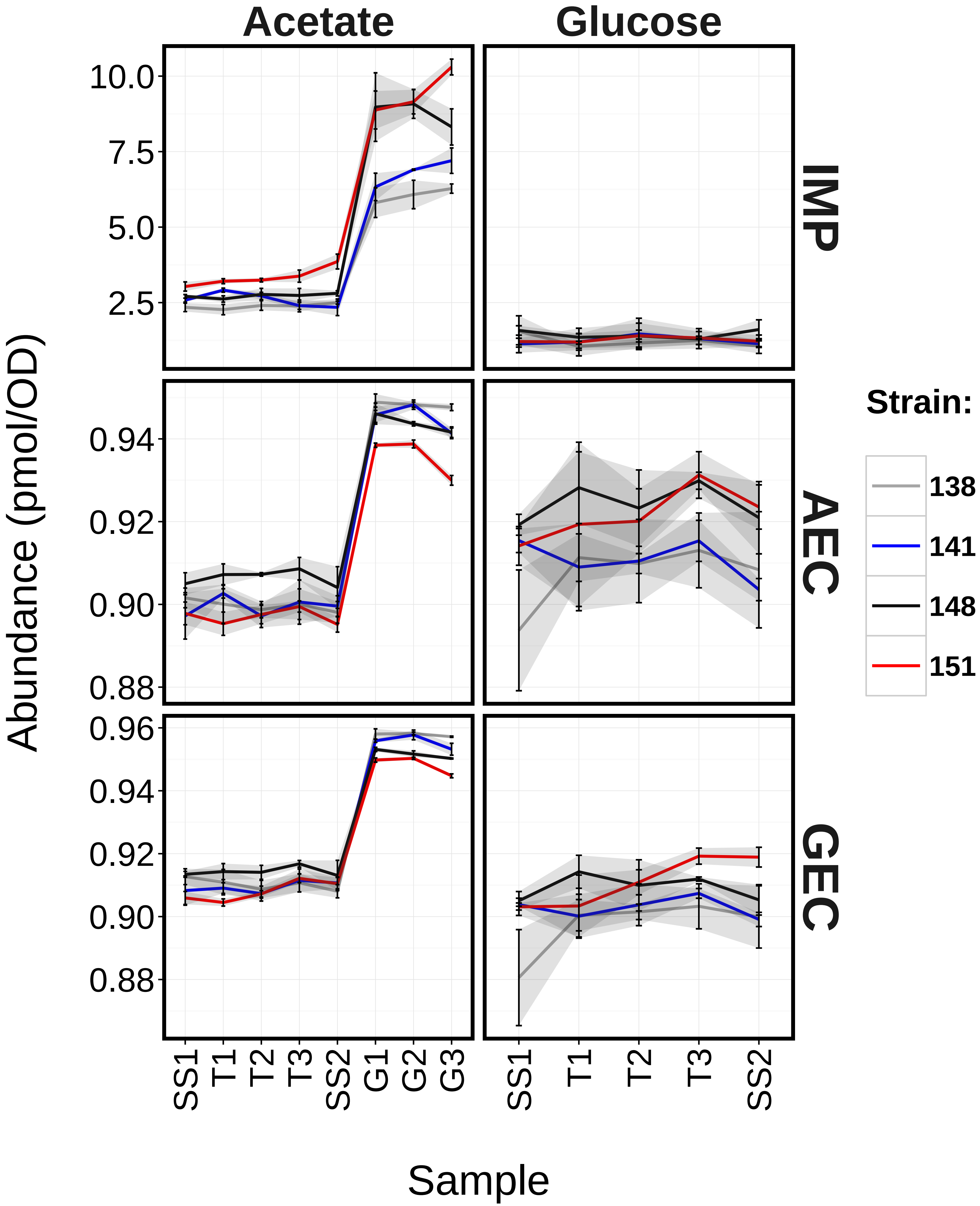

Acetate
Glucose
IMP
AEC
GEC
Strain:
138
141
148
151
Abundance (pmol/OD)
Sample
10.0
7.5
5.0
2.5
0.94
0.92
0.90
0.88
0.96
0.94
0.92
0.90
0.88
SS1 T1 T2 T3 SS2 G1 G2 G3
SS1 T1 T2 T3 SS2
